# Interaction of lipoprotein QseG with sensor kinase QseE in the periplasm controls the phosphorylation state of the two-component system QseE/QseF in *Escherichia coli*

**DOI:** 10.1371/journal.pgen.1007547

**Published:** 2018-07-24

**Authors:** Yvonne Göpel, Boris Görke

**Affiliations:** Department of Microbiology, Immunobiology and Genetics, Max F. Perutz Laboratories (MFPL), University of Vienna, Vienna Biocenter (VBC), Vienna, Austria; Institut Pasteur, CNRS UMR 3525, FRANCE

## Abstract

Histidine kinase QseE and response regulator QseF compose a two-component system in *Enterobacteriaceae*. In *Escherichia coli* K-12 QseF activates transcription of *glmY* and of *rpoE* from Sigma 54-dependent promoters by binding to upstream activating sequences. Small RNA GlmY and RpoE (Sigma 24) are important regulators of cell envelope homeostasis. In pathogenic *Enterobacteriaceae* QseE/QseF are required for virulence. In enterohemorrhagic *E*. *coli* QseE was reported to sense the host hormone epinephrine and to regulate virulence genes post-transcriptionally through employment of GlmY. The *qseEGF* operon contains a third gene, *qseG*, which encodes a lipoprotein attached to the inner leaflet of the outer membrane. Here, we show that QseG is essential and limiting for activity of QseE/QseF in *E*. *coli* K-12. Metabolic ^32^P-labelling followed by pull-down demonstrates that phosphorylation of the receiver domain of QseF *in vivo* requires QseE as well as QseG. Accordingly, QseG acts upstream and through QseE/QseF by stimulating activity of kinase QseE. ^32^P-labelling also reveals an additional phosphorylation in the QseF C-terminus of unknown origin, presumably at threonine/serine residue(s). Pulldown and two-hybrid assays demonstrate interaction of QseG with the periplasmic loop of QseE. A mutational screen identifies the Ser58Asn exchange in the periplasmic loop of QseE, which decreases interaction with QseG and concomitantly lowers QseE/QseF activity, indicating that QseG activates QseE by interaction. Finally, epinephrine is shown to have a moderate impact on QseE activity in *E*. *coli* K-12. Epinephrine slightly stimulates QseF phosphorylation and thereby *glmY* transcription, but exclusively during stationary growth and this requires both, QseE and QseG. Our data reveal a three-component signaling system, in which the phosphorylation state of QseE/QseF is governed by interaction with lipoprotein QseG in response to a signal likely derived from the cell envelope.

## Author summary

Bacteria use two-component systems, composed of a membrane-bound histidine kinase and a cytoplasmic response regulator, to sense environmental cues and adapt gene expression accordingly. The enterobacterial two-component system QseE/QseF controls functions related to the cell envelope. In pathogens, QseE/QseF were suggested to regulate virulence in response to the host hormone epinephrine, a process known as interkingdom signaling. Here, we analyzed the role of *qseG*, which co-localizes with *qseE/qseF* and encodes a periplasmic lipoprotein. We show that QseG is a prerequisite for QseE/QseF activity in *E*. *coli* K-12. Without QseG, kinase QseE is unable to phosphorylate and activate response regulator QseF. Furthermore, QseG and QseE interact in the periplasm and a mutation in QseE impairing interaction concomitantly decreases activity. Our data reveal a regulatory cascade likely conserved in other *Enterobacteriaceae*, in which membrane-bound QseE is stimulated through interaction with periplasmic QseG to phosphorylate cytoplasmic QseF. Finally, we show that epinephrine is a minor stimulus for QseE/QseF in *E*. *coli* K-12 and that its sensing strictly depends on QseG. Therefore, QseG is not only required for activity of QseE/QseF, but also for signal perception.

## Introduction

Two-component systems (TCSs) allow bacteria to perceive information from the environment and to adapt gene expression and behavior in a meaningful way. Typically, a membrane-bound histidine kinase senses a stimulus via its N-terminal input domain leading to auto-phosphorylation at a histidine residue in the C-terminal transmitter domain [1]. Subsequently, the phosphoryl-group is transferred to an aspartate residue in the receiver domain of the cognate response regulator, thereby activating the associated output domain, which is often a transcription factor. While the downstream functions of many TCSs are well characterized, the stimuli sensed by the kinases and the underlying mechanisms often remain elusive. Histidine kinases may perceive their cognate stimuli directly or through employment of accessory proteins [2–5]. The model organism *E*. *coli* K-12 encodes 29 TCSs, each dedicated to a specific function [6, 7]. Albeit intensively investigated, the roles of some TCSs still remain weakly defined including the TCS QseE/QseF (a.k.a. GlrK/GlrR, a.k.a. YfhK/YfhA), which is conserved in *Enterobacteriaceae* [8].

**Fig 1:**
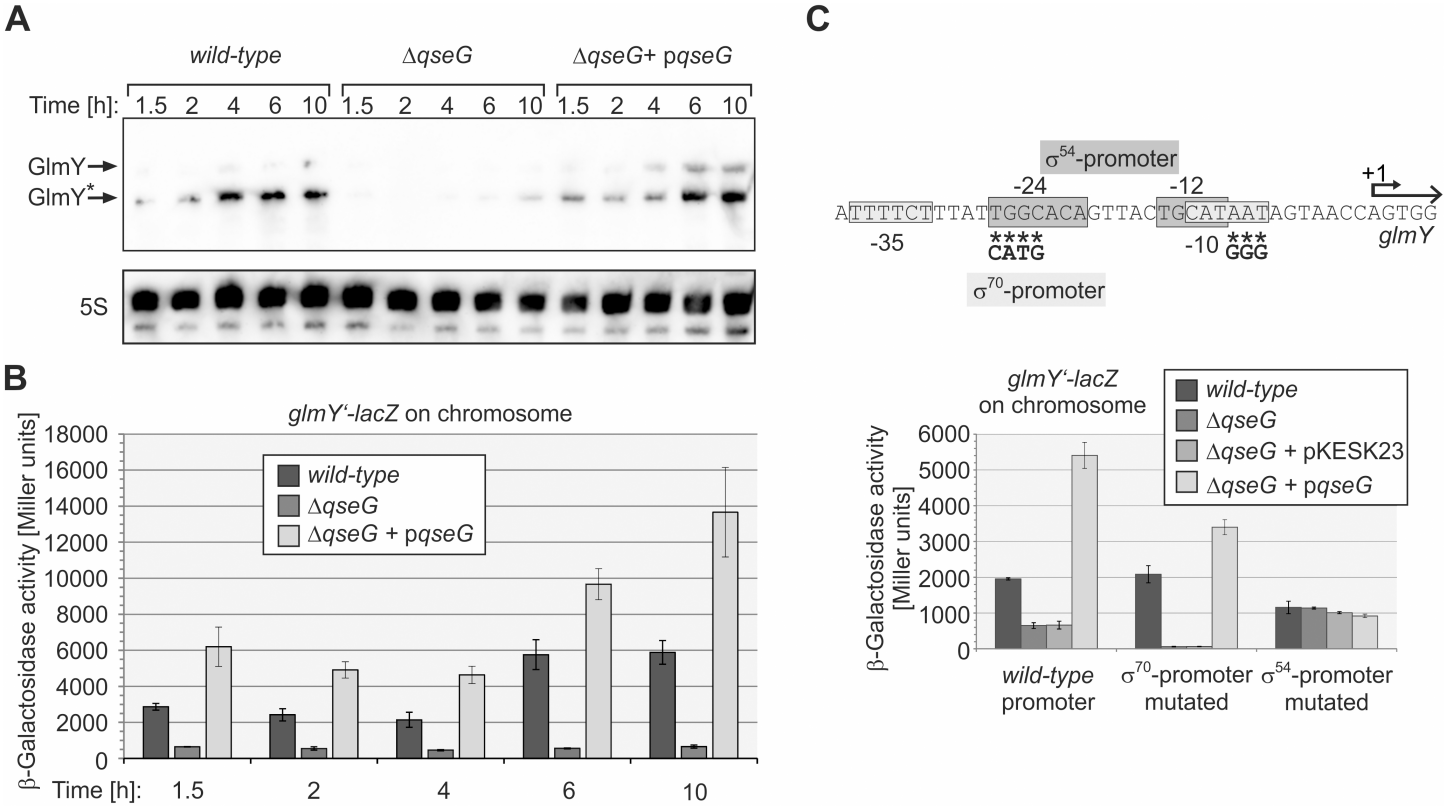
QseG is required for expression of sRNA GlmY. **A**. Northern Blot assessing abundance of GlmY in *E*. *coli wild-type* strain Z197, the *ΔqseG* mutant strain Z477 and strain Z477 carrying plasmid pBGG225, which transcribes *qseG* from the *P*_*Ara*_ promoter. Cells were grown in LB and total RNA was isolated at the indicated time intervals and analyzed by Northern blotting using a probe directed against GlmY. Full-length and processed GlmY species are indicated by arrows, the 5S rRNA loading control is provided in the lower panel. **B)** β-Galactosidase activities of strains carrying a *glmY’-lacZ* reporter fusion in the *λattB* site on the chromosome: Z197 (*wild-type*), Z477 (Δ*qseG*) and Z477 carrying *qseG* on plasmid pYG220 under control of the *P*_*tac*_ promoter. Cells were grown in LB and β-galactosidase activities were assayed at the indicated time of growth. Corresponding growth curves are presented in S2A Fig. **C)** Schematic representation of the *glmY* promoter region (top panel). -35/-10 motifs for σ^70^ are boxed in light grey, -24 and -12 binding sequences for σ^54^ are boxed in dark grey and the transcriptional start site is designated +1. Asterisks denote mutated nucleotides; the introduced changes are shown below. Bottom panel: β-Galactosidase activities were determined in the following strains and transformants (left to right): Z197, Z477, Z477/pKESK23 (empty vector control), Z477/pYG220 (encoding *qseG*) in context of the *wild-type* promoter; Z190, Z449, Z449/pKESK23, Z449/pYG220 in context of the mutated σ^70^ promoter and Z201, Z492, Z492/pKESK23, Z492/pYG220 in context of the mutated σ^54^ promoter.

Response regulator QseF comprises an N-terminal receiver domain, a σ^54^ interaction domain and a C-terminal DNA-binding helix-turn-helix (H-T-H) motif. In *E*. *coli* K-12, QseF activates σ^54^-dependent promoters located upstream of genes *glmY* and *rpoE*, respectively [9, 10]. GlmY is a small RNA (sRNA) controlling cell envelope biosynthesis (see below) and *rpoE* encodes σ^24^, a master regulator of the cell envelope stress response [11, 12]. The *glmY* gene is located immediately upstream of the *qseEGF* operon encoding the QseE/QseF TCS and this synteny is conserved [8, 13]. Assisted by the integration host factor, QseF binds three upstream activating sequences (UAS) with the consensus TGTN_12_ACA thereby triggering transcription of *glmY* from its σ^54^-promoter [8, 9]. An overlapping weak σ^70^-promoter contributes to low basal expression levels (Fig 1C; [8]). UAS similar to those present upstream of *glmY* are also observed upstream of the recently identified σ^54^-dependent *rpoE* P2 promoter shown to be activated by QseF [10]. QseF requires phosphorylation by kinase QseE for activity. Phosphorylation of QseF increases its DNA-binding affinity and activity of the *glmY* σ^54^-promoter is abolished in mutants lacking QseE [8, 9]. QseE/QseF is one of few TCSs residing in the “on” state, at least partially, which is in contrast to many other TCSs, which require a specific signal for activation that is usually absent from standard laboratory conditions.

GlmY has a crucial role for the bacterial cell: Together with the homologous sRNA GlmZ and the RNA-binding adaptor protein RapZ, it controls the levels of enzyme GlmS, which synthesizes glucosamine-6-phosphate, an essential precursor for peptidoglycan and the outer membrane [11]. GlmZ is an Hfq-dependent sRNA and base-pairs with the *glmS* transcript, thereby stimulating translation and stabilizing the mRNA [14, 15]. When not required, GlmZ is recruited by RapZ to degradation by RNase E [16–18]. The latter process is counteracted by sRNA GlmY, which accumulates when the intracellular glucosamine-6-phosphate concentration decreases [16, 19]. GlmY is not an Hfq-binding sRNA [17]. It acts as decoy RNA and serves to sequester RapZ, thereby inhibiting decay of GlmZ, which then stimulates GlmS production to replenish glucosamine-6-phosphate [16]. This feedback mechanism also operates in *Salmonella* and perhaps in all *Enterobacteriaceae* ensuring homeostasis of cell envelope precursors [20]. Accumulation of GlmY in response to glucosamine-6-phosphate depletion occurs post-transcriptionally indicating that QseE/QseF are not sensing this metabolite [9]. Thus, the stimulus for the QseE/QseF TCS in *E*. *coli* K-12 remains unknown so far.

In addition to its crucial function in governing expression of cell envelope regulators, the QseE/QseF TCS is required for virulence of pathogenic *Enterobacteriaceae*. As a common theme, deletion mutants of the *qseEGF* operon are attenuated in virulence, as demonstrated for *Citrobacter rodentium*, the fish pathogen *Edwardsiella tarda*, enterohemorrhagic *Escherichia coli* (EHEC), *Salmonella enterica* and *Yersinia pseudotuberculosis* [21–25]. This phenomenon has been studied most thoroughly in EHEC, in which QseE/QseF together with the QseB/QseC TCS complexly regulate virulence genes encoded within and outside of the locus of enterocyte effacement (LEE), a pathogenicity island organized in 5 operons (for recent reviews, see: [26–28]). Regulation by QseE/QseF is indirect and occurs through GlmY/GlmZ, which promote translation of virulence gene *espFU* and selectively destabilize transcripts of the LEE4 and 5 operons [29]. Kinase QseC is a sensor of epinephrine (Epi) and norepinephrine in EHEC [30] and the QseE/QseF TCS has been described to participate in Epi sensing and signal transduction involving extensive cross-talk between both TCSs [29, 31]. EHEC and also other bacteria including *C*. *rodentium* and *Salmonella* sense these host hormones to activate virulence gene expression and colonize the host [26]. Epi was shown to stimulate autophosphorylation of QseC as well as QseE *in vitro* [30, 31]. In addition, QseF can also be cross-phosphorylated by the non-cognate kinase QseC *in vitro* [32]. By integration of these phosphorylation signals QseF is proposed to modulate expression of virulence genes in response to Epi [33]. Whether Epi also plays a role for activity of QseE/QseF in commensal bacteria is unknown.

The operon encoding the QseE/QseF TCS contains a third gene, *qseG*. QseG carries an N-terminal signal sequence recognized by the general Sec secretory pathway or the Tat twin arginine translocation system. Consistently, in EHEC QseG was shown to reside in the outer membrane facing the periplasmic leaflet [24, 31]. In pathogenic bacteria including EHEC, *C*. *rodentium* and *Salmonella*, *qseG* is required for virulence and host colonization, but the underlying mechanisms remain unclear [24]. In the current work, we investigated the role of QseG in *E*. *coli* K-12. We show that QseG is essential for activity of the QseE/QseF TCS and thereby for *glmY* transcription. *In vivo* phosphorylation assays demonstrate that QseG is mandatory for kinase QseE activity and thereby for QseF phosphorylation. We show that QseG interacts with the periplasmic domain of kinase QseE and mutational analysis indicates that this interaction is required for QseE activity. Finally, we show that Epi slightly increases QseF phosphorylation and thereby *glmY* expression in a QseE- and QseG-dependent manner in the stationary growth phase. Taken together, our data show that QseG operates together with QseE/QseF constituting a three-component system. QseG is likely involved in sensing of the cognate stimulus.

## Results

### The cell envelope protein QseG is indispensable for activity of the σ^54^-promoter directing expression of *glmY*

First, we confirmed that QseG is present in the periplasmic space in *E*. *coli* K-12. To this end, we isolated the *E*. *coli* cell envelope containing periplasmic and outer membrane proteins using an extraction method, which was shown to produce clean envelope extracts [34]. To allow for its detection, QseG was provided with a C-terminal Strep-tag epitope. *E*. *coli* cells carrying a plasmid encoding *qseG-strep* or the empty expression vector were grown to the exponential as well as to the stationary growth phase. Western blot analysis of total protein extracts confirmed proper synthesis of QseG-Strep (S1 Fig, top panel, lanes 1–4). Envelope extracts were prepared and analyzed by SDS-PAGE and Western blotting. Comparison of the protein bands revealed a distinctive pattern of the periplasmic extracts as compared to the total extracts, indicating successful fractionation (S1 Fig, bottom panel). Indeed, the periplasmic maltose binding protein (MBP; MW = 43.39 kDa) could be readily detected in the envelope extracts, whereas the cytoplasmic ribosomal protein S1 (MW = 61.16 KDa) was absent, confirming successful isolation of cell envelope proteins (S1 Fig, panels 2 and 3). Western analysis detected QseG-Strep in the envelope extracts and this localization was unaffected by the growth stage (S1 Fig, top panel, lanes 7 and 8). In conclusion, QseG is present in the cell envelope of *E*. *coli* K-12, in agreement with previous results in EHEC [31].

To address the role of QseG for activity of the QseE/QseF TCS, we first studied the impact of a *qseG* deletion on GlmY steady state levels. Total RNA was extracted from bacteria harvested at various times during growth and analyzed by Northern blotting. In the *wild-type* strain GlmY accumulated over time showing highest levels during transition to the stationary growth phase, recapitulating previous observations (Fig 1A; [9, 16]). In the *ΔqseG* strain, GlmY levels were drastically decreased albeit weak hybridization signals remained detectable. GlmY levels were perfectly restored upon introduction of a plasmid expressing *qseG* from a heterologous promoter, excluding negative interference of the *qseG* deletion with synthesis of the downstream encoded response regulator QseF (Fig 1A). To determine whether QseG affects GlmY levels at the transcriptional or post-transcriptional level, we measured expression of an ectopic *glmY’-lacZ* reporter fusion integrated into the chromosomes of the respective strains (Fig 1B). Expression of the *glmY’-lacZ* fusion increased over time in the *wild-type* strain, whereas only low activities were detectable in the *ΔqseG* mutant. Complementation of the *ΔqseG* mutant with a multi-copy plasmid expressing *qseG* from a heterologous promoter restored *glmY’-lacZ* expression to levels that exceeded the activities measured in the *wild-type* (Fig 1B). The requirement of *qseG* for *glmY* transcription was not only detectable in strain CSH50 derivatives, which were used here, but also in MG1655, indicating that this is a general phenomenon affecting *E*. *coli* K-12 strains (S3 Fig). In conclusion, QseG is required for efficient transcription of *glmY*.

Next, we dissected whether QseG controls the σ^54^- or the σ^70^- or both promoters upstream of *glmY*. To this end, we used mutated reporter gene fusions carrying nucleotide exchanges in the *glmY* transcriptional control region, which abolish activity of one promoter while leaving the respective second promoter unaffected (Fig 1C top). We determined the activities of these reporter constructs in exponentially growing *wild-type* and *ΔqseG* strains (Fig 1C bottom). Expression of the fusion solely driven by the σ^54^-promoter was abolished in the *ΔqseG* mutant and perfectly restored upon complementation with a plasmid carrying *qseG*. Complementation was observed regardless whether *qseG* was expressed from an IPTG-inducible or an arabinose-inducible expression vector (Fig 1C and S4 Fig). In contrast, activity of the σ^70^-promoter was unaffected by *qseG* deletion or overexpression (Note that basal expression levels are elevated in this case, because the σ^70^-promoter is usually repressed by binding of σ^54^ to the overlapping σ^54^-promoter; [9]). Hence, *qseG* is essential for activity of the σ^54^-promoter of *glmY*, but has no role for the σ^70^-promoter, explaining the low residual expression of *glmY* that remained detectable in the *qseG* deletion mutant (Fig 1A–1C). These results strongly resemble previous data obtained in a mutant lacking kinase QseE [9], i.e. the Δ*qseG* allele phenocopies a Δ*qseE* mutation. As the σ^54^-promoter of *glmY* is controlled by QseE/QseF, one likely explanation for these results is that QseG has a role for activity of this TCS.

### QseG triggers *glmY* expression through the QseE/QseF TCS

**Fig 2:**
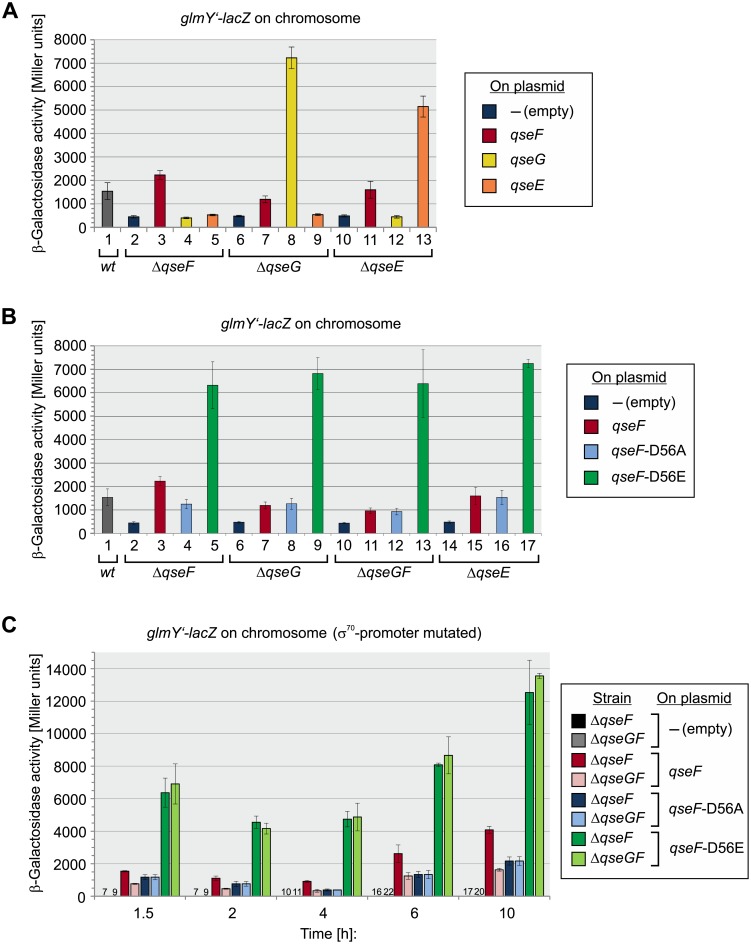
QseG is required for QseE/QseF activity. **A**. QseG acts upstream of QseF and requires QseE and QseF to enhance *glmY* transcription. Strains Z197 (*wild-type*, column 1), Z206 (Δ*qseF*, columns 2–5), Z477 (Δ*qseG*, columns 6–9) and Z970 (Δ*qseE*, columns 10–13) were addressed, which carry a *glmY’-lacZ* fusion on the chromosome. The strains carried the following plasmids expressing the indicated genes: pKESK23 (empty plasmid; dark blue bars), pYG89 (*qseF*, red bars), pYG220 (*qseG*, yellow bars), pYG221 (*qseE*, orange bars). The β-galactosidase activities produced by exponentially growing cells are reported. **B**. Activity of QseF variants in Δ*qseG*, Δ*qseGF* and Δ*qseE* mutants. β-Galactosidase activities produced by Δ*qseF* (Z206, columns 2–5), Δ*qseG* (Z477, columns 6–9), Δ*qseGF* (Z922, columns 10–13) and Δ*qseE* (Z970, columns 14–17) strains carrying a chromosomal *glmY’-lacZ* fusion. These strains either harbored an empty plasmid (pKESK23, dark blue bar), or plasmids expressing *wild-type qseF* (pYG89, red bar), *qseF*-D56A (pYG93, light blue bar) or *qseF*-D56E (pYG90, green bar). For comparison, the *wild-type* strain Z197 is shown (column 1). β-Galactosidase activities were determined from cells growing exponentially in LB medium. **C**. QseG stimulates activity of *wild-type* QseF, while leaving QseF mutants carrying exchanges of the D56 phosphorylation unaffected. Strains Z196 (Δ*qseF*, dark bars) and Z955 (Δ*qseGF*, light bars) carry a chromosomal *glmY’-lacZ* fusion in context of the mutated σ^70^-promoter (see Fig 1C). The strains harbored the following plasmids: pKESK23 (empty vector, black and grey columns; note that due to low activities these columns are not visible in the graph), pYG89 (*qseF*, dark and light red columns), pYG93 (*qseF*-D56A, dark and light blue columns), pYG90 (*qseF*-D56E, dark and light green columns). β-Galactosidase activities were determined during growth in LB medium at the indicated times. The corresponding growth curves are shown in S2B Fig. The β-galactosidase activities of strains carrying the empty plasmid pKESK23 are expressed as numerical values.

To determine whether QseG acts up- or downstream of QseE/QseF on *glmY*, we performed epistasis experiments. To this end, we tested the effects of plasmid-driven *qseF*, *qseG* and *qseE* overexpression in *qse* deletion mutants. In absence of *qseF*, transcription of *glmY’-lacZ* is solely driven by the σ^70^-promoter and therefore significantly decreased as compared to the *wild-type* strain (Fig 2A, compare columns 1 and 2; [9]). Similar low expression levels were observed in the *ΔqseG* and *ΔqseE* mutants confirming that QseE and QseG are required for activity the σ^54^ *glmY* promoter (Fig 2A, columns 6 and 10). Complementation of the various deletion mutants with corresponding genes on plasmids restored high *glmY’-lacZ* expression levels, ruling out impaired expression of the remaining *qse* genes in the individual deletion mutants (Fig 2A, columns 3, 8, 13). Notably, plasmid-driven overexpression of *qseG* in the *ΔqseF* and *ΔqseE* mutants had no effect on the weak *glmY* expression level (Fig 2A, columns 4 and 12). Likewise, overexpression of *qseE* was without any effect when tested in the *ΔqseF* and *ΔqseG* mutants (Fig 2A, columns 5 and 9). These data show that QseG requires both QseF as well as QseE to stimulate *glmY* expression. Moreover, QseE is apparently unable to stimulate QseF activity when QseG is absent. Interestingly, when the *qseG* expression plasmid was used to complement the *ΔqseG* mutant strain, a very high *glmY* expression level was observed, suggesting that QseG is limiting for QseF activity in the *wild-type* strain (Fig 2A, columns 1 and 8). This conclusion is further supported by an experiment in which *qseG* was transcribed from the arabinose-inducible *P*_*Ara*_ promoter on a low copy plasmid and expression was gradually increased using incremental arabinose concentrations. A concomitant increase of *glmY* expression was observable indicating that *glmY* promoter activity directly correlates with the QseG level (S5 Fig).

To learn whether QseG has a role for QseF phosphorylation, we studied the effect of QseG on QseF variants carrying mutations in the D56 phosphorylation site, i.e. QseF-D56A and QseF-D56E variants mimicking non-phosphorylated and phosphorylated QseF, respectively [8]. Plasmids encoding *wild-type* QseF and the mutant QseF variants were used to complement strains deleted for chromosomal *qseF*, *qseG* or both genes, respectively. Introduction of the plasmid encoding *wild-type* QseF into the *ΔqseF* mutant restored *glmY’-lacZ* levels above the levels observed in the *wild-type* strain (Fig 2B, compare columns 1–3). Complementation of the *ΔqseF* mutant with the plasmid encoding the phospho-ablative QseF-D56A variant resulted in activities, which were 2-fold lower but clearly above background levels (Fig 2B, compare columns 2–4). It is well-known that upon overproduction even non-phosphorylated response regulators are able to activate their target genes to some extent [4, 35, 36] and this also applies to QseF [8]. In contrast, introduction of the plasmid coding for the phospho-mimetic QseF-D56E variant generated a much higher *glmY* expression level (Fig 2B, column 5), confirming that phosphorylated QseF is active and strongly stimulates *glmY* expression [8]. We observed very similar *glmY* expression patterns when the various *qseF* expression plasmids were tested in *ΔqseG*, *ΔqseGF* and *ΔqseE* mutant strains, but there was one striking exception: In the latter mutants, comparable activities were produced by wild-type QseF and the non-phosphorylatable QseF-D56A variant, respectively. In contrast, when tested in the *ΔqseF* mutant two-fold higher activities were generated by plasmid-borne wild-type QseF as compared to QseF-D56A (Fig 2B, compare columns 3–4 with 7–8, 11–12 and 15–16). These observations suggest that QseG, just as QseE, can stimulate the activity of *wild-type* QseF, but not of QseF variants bearing exchanges in the D56 phosphorylation site.

To investigate the role of QseG for QseF activity in more detail, we compared the activities of the various plasmid-encoded QseF variants in isogenic *ΔqseF* and *ΔqseGF* strains during growth. In this case, we used a *glmY’-lacZ* fusion solely driven from the σ^54^-promoter and monitored β-galactosidase activities at regular time intervals (Fig 2C). Expression of the phospho-mimetic *qseF*-D56E variant resulted in very high *glmY* expression levels, whereas much lower activities were measured when the phospho-ablative *qseF*-D56A mutant was expressed (Fig 2C, compare blue and green columns). Of note, presence or absence of *qseG* had no role for the activities generated by these *qseF* alleles. In contrast, *glmY* expression levels triggered by *wild-type* QseF always decreased in the absence of *qseG* to the levels observed for the phospho-ablative QseF-D56A variant (Fig 2C, compare dark red with light red and blue columns). Taken together, these data suggest that QseG stimulates activity of response regulator QseF in a dosage-dependent manner, most likely by triggering its phosphorylation.

### Absence of transcriptional autoregulation of the *qseEGF* operon

**Fig 3:**
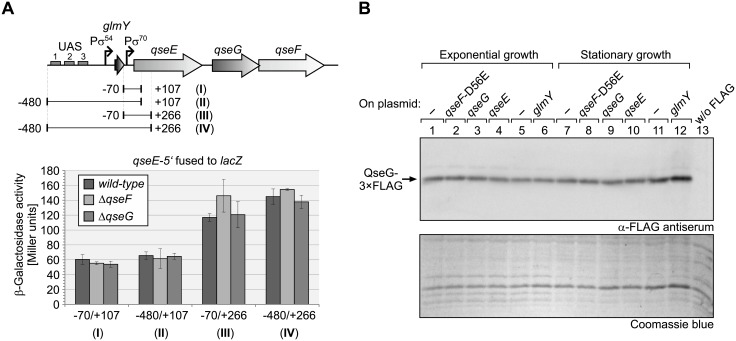
The *qseEGF* operon is not subject to transcriptional autoregulation. **A**. Expression of transcriptional *qseE’-lacZ* reporter fusions is not affected by deletion of *qseF* or *qseG*. The *glmY* locus and the adjacent *qseEGF* operon are schematically depicted at the top. Experimentally verified promoters directing expression of *glmY* and *qseEGF*, respectively [9], are indicated by arrows. For reporter gene studies, the regions indicated by horizontal lines and roman numerals were fused to *lacZ*. Positions are relative to the first nucleotide of the *qseE* start codon. The *lacZ* fusions were placed on plasmids pBGG273 (fusion I), pBGG274 (fusion II), pBGG354 (fusion III), pBGG355 (fusion IV) and subsequently introduced into strains R1279 (wild-type), Z179 (Δ*qseF*) and Z1117 (Δ*qseG*), respectively. The β-galactosidase activities of these transformants were determined from exponentially growing cells (bottom) as well as from stationary cells (S6 Fig). **B**. Plasmid-driven over-expression of *qseF*-D56E, *qseG*, *qseE* or *glmY* does not affect the level of chromosomally encoded QseG. Strain Z951 was addressed, which carries the sequence coding for the 3×FLAG epitope fused in frame to the 3’ end of *qseG* encoded at its natural locus in the chromosome. In addition, strain Z951 carried the following plasmids overproducing the indicated genes, respectively: pKESK23 (empty vector control for *qse* plasmids; lanes 1, 7), pYG90 (*qseF*-D56E; lanes 2, 8), pYG220 (*qseG*; lanes 3, 9), pYG221 (*qseE*; lanes 4, 10), pBR-plac (empty vector control for *glmY* plasmid; lanes 5, 11), pYG83 (*glmY*; lanes 6, 12). As a negative control, strain R1279 lacking a FLAG epitope was tested in lane 13. The various transformants were grown in LB and total protein extracts, prepared from cells harvested in the exponential as well as in the stationary growth phase, were analyzed by Western blotting using α-FLAG antiserum (top panel). As a loading control, the Coomassie blue stained PAA gel is shown in the bottom panel.

Several two-component systems are known, which are subject to autoregulation at the transcriptional level [5]. A putative autoregulation could potentially interfere with our genetic analysis addressing the role of QseG for QseE/QseF activity. To investigate a possible feedback regulation, we measured expression of ectopic transcriptional *lacZ* fusions to the *qseEGF* promoter. The *qseEGF* operon is transcribed from a σ^70^-promoter, which is located immediately downstream of the *glmY* gene, and starts transcription 25 bp upstream of the *qseE* start codon (Fig 3A top; [9]). A fusion of *lacZ* to a DNA fragment comprising this promoter (position -70 to +107 relative to the *qseE* start), generated only low β-galactosidase activities that were not affected by *qseF* and *qseG* mutations (Fig 3A, fusion I). To account for potential *glmY*-*qseE* read-through transcripts, a fusion of *lacZ* to a fragment comprising positions -480 to +107 relative to *qseE* was additionally tested. The latter fusion carried the complete *glmY* locus including its transcriptional control region upstream of the *qseE* promoter and *qseE’*-*lacZ* (Fig 3A, fusion II). However, the activities generated by this construct were virtually indistinguishable from the activities observed for the shorter fusion (Fig 3A, compare fusions I and II). To account for a possible intrinsic instability of the *qseE’* (+107)-*lacZ* fusion mRNAs, we additionally tested isogenic constructs, in which the *lacZ* gene was fused further downstream at position +266 to *qseE* (Fig 3A, fusions III and IV). Indeed, these fusions generated somewhat higher activities as compared to fusions I and II, but once again activities were not affected by deletion of *qseF* or *qseG* or presence of the *glmY* locus upstream of *qseE’* (+266)-*lacZ*. Similar expression patterns were observed in the exponential and stationary growth phases (compare Fig 3A and S6 Fig). These results argue against an autoregulation of *qseEGF* expression. Moreover, the data are in agreement with previous Northern results indicating that *qseE* is only weakly expressed, and with previous semi-quantitative RT-PCR data suggesting that read-through from the upstream located *glmY* promoter into *qseE* does virtually not occur [9]. Low expression of *qseE* is also reflected by weak signals obtained for FLAG-tagged QseE in Western blot analyses of total protein extracts as shown later in this study. In agreement, a global proteomics study measured 11 molecules QseG and 36 molecules QseF per *E*. *coli*-K12 cell, whereas QseE could not be detected at all [37].

To corroborate these data and to account for a hypothetical internal promoter that could be present in the *qseE*-*qseG* intergenic region, we additionally monitored the levels of endogenously encoded QseG protein. To allow for detection, the 3×FLAG epitope sequence was fused to the 3’ end of the chromosomal *qseG* gene. Reporter gene measurements confirmed that the QseG-3×FLAG protein retained full functionality in respect to activation of *glmY* transcription (S7 Fig). To test for autoregulation, we refrained from analysis of deletions within the *qseEGF* operon as this procedure would generate shorter *qse* transcripts with likely altered stabilities, thereby leading to ambiguous results. In lieu thereof, we tested the effects of plasmid-driven over-expression of *qseF*-D56E, *qseG* and *qseE* on synthesis of the chromosomally encoded QseG-3×FLAG protein, respectively. The same plasmids trigger a strong expression of the *glmY’-lacZ* reporter fusion (Fig 2), reflecting the properties of a fully activated QseE/QseF TCS. Since downstream effectors are sometimes involved in feedback regulation of two-component systems [5], a plasmid overexpressing *glmY* was included in this analysis. However, none of the tested plasmids had any significant effect on the QseG-3×FLAG level, neither during exponential growth nor in the stationary growth phase (Fig 3B). In conclusion, QseG controls activity of QseE/QseF rather than expression of corresponding genes.

### Response regulator QseF is phosphorylated at multiple sites *in vivo*

**Fig 4:**
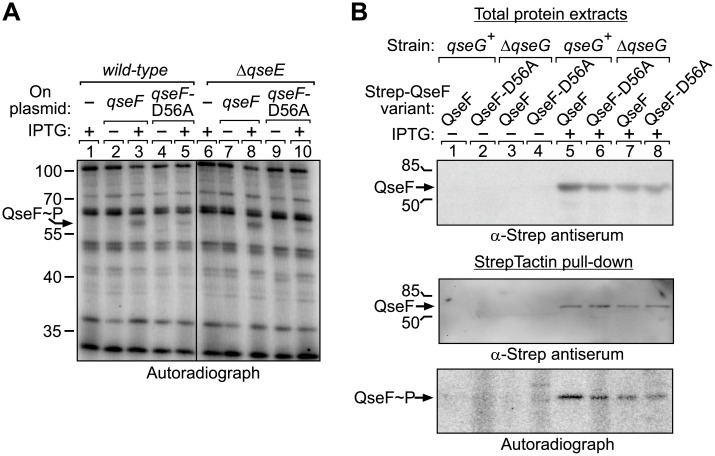
QseF is phosphorylated at multiple sites *in vivo*. **A**. *In vivo* H_3_[^32^P]O_4_ labeling of strains Z197 (*wild-type*) and Z970 (Δ*qseE*) carrying either the empty expression vector pKES170 (lanes 1, 6) or overexpressing *wild-type qseF* from plasmid pYG253 (lanes 2–3, 7–8) or *qseF*-D56A from plasmid pYG254 (lanes 4–5, 9–10). For induction of *qseF* expression IPTG was added as indicated. After metabolic [^32^P] labeling total protein extracts were analyzed by SDS-PAGE and autoradiography. **B**. StrepTactin pull-down assay of QseF after metabolic [^32^P] labeling. Strains Z196 (Δ*qseF*, *qseG*^+^) and Z955 (Δ*qseGF*) were transformed with plasmids pYG269, expressing *qseF-strep* and pYG269-D56A encoding *qseF-D56A-strep*. Transformants were grown to mid log phase (OD_600_ ~0.8), 1 mM IPTG was added where indicated and whole cell extracts were analyzed by western blotting using α-Strep antiserum (input, top). H_3_[^32^P]O_4_ metabolic labeling was followed by StrepTactin pull-down and pull-down fractions were analyzed by Western blotting with α-Strep antiserum (middle) and by SDS-PAGE and autoradiography (bottom).

The genetic data (Fig 2) point to a mechanism in which QseG stimulates phosphorylation of response regulator QseF through modulation of activity of kinase QseE. To address this possibility, we studied phosphorylation of QseF *in vivo* by metabolic labeling of cells using [^32^P] phosphorus. To this end, *qseF* was expressed under control of the IPTG-inducible *P*_*tac*_ promoter from a plasmid in *wild-type* as well as *ΔqseE* cells. The QseF variant carrying the D56A exchange of the phosphorylation site in the receiver domain served as negative control. The bacteria were grown in absence and presence of IPTG and subsequently labeled with [^32^P] phosphoric acid. Total protein extracts were separated by SDS-PAGE and analyzed by autoradiography (Fig 4A). Among various bands representing abundant phosphoproteins, a single phosphorylation signal became visible exclusively in the presence of IPTG and its position on the gel roughly matched the molecular weight of QseF (MW = 49.15 kDa). To our surprise, this phosphorylation signal was also detectable in the *ΔqseE* strain and when the QseF-D56A variant was employed. To confirm that the IPTG-inducible phosphorylation signal indeed corresponds to QseF, we used QseF variants carrying Strep-tags at their C-termini allowing for their pull-down following [^32^P] labeling. In this case, the QseF variants were produced from plasmids in *ΔqseF* (*qseG*^+^) as well as in *ΔqseFG* cells (Fig 4B, top panel), metabolically labeled and subsequently isolated by pull-down using StrepTactin coated magnetic beads. The obtained fractions were separated by SDS-PAGE and analyzed by Western blotting using anti-Strep antiserum as well as by autoradiography (Fig 4B, middle and bottom panels). The Western blot confirmed successful isolation of the QseF proteins from the cultures induced by IPTG (Fig 4B middle panel, lanes 5–8), whereas QseF could not be recovered from non-induced cells (Fig 4B middle panel, lanes 1–4). Autoradiography once again detected phosphorylation of QseF under all conditions, regardless of the D56A substitution and also not affected by QseG (Fig 4B bottom panel, lanes 5–8).

**Fig 5:**
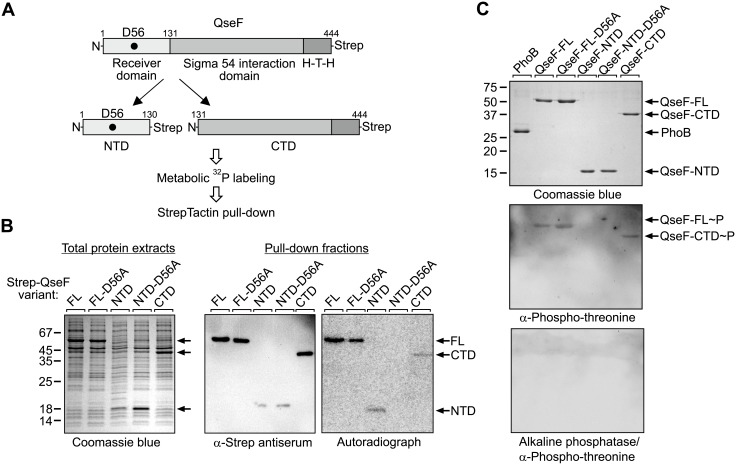
QseF is phosphorylated *in vivo* at Asp56 in the receiver domain and at an additional site in the C-terminus. **A**. Schematic representation of C-terminally Strep-tagged QseF and the various truncations used for metabolic labeling and StrepTactin pull-down. QseF comprises an N-terminal receiver domain, a σ^54^ interaction domain and C-terminal helix-turn-helix (H-T-H) DNA-binding domain. Location of the conserved D56 phosphorylation site in the receiver domain is indicated by a black dot. **B**. StrepTactin pull-down assay of truncated QseF variants following metabolic [^32^P] labeling. Strain Z196 (Δ*qseF*) was employed, which harbored the following plasmids encoding the Strep-tagged QseF variants given in parentheses, respectively: pYG278 (FL = full-length QseF), pYG278-D56A (FL-D56A = full-length QseF with D56A exchange), pYG279 (NTD), pYG279-D56A (NTD-D56A) and pYG280 (CTD). Induction of synthesis of recombinant proteins following addition of IPTG was checked by separation of total protein extracts by SDS-PAGE and subsequent Coomassie blue staining (left panel). Cells were labelled with [^32^P] and Strep-tagged proteins were isolated by pull-down and subsequently analyzed by Western Blotting using an antibody directed against the Strep-tag (middle panel) and by autoradiography (right panel). **C**. Western blot addressing the nature of the phosphorylation of the QseF-CTD. Three μg each of the purified Strep-tagged proteins indicated in the figure were separated by 15% SDS-PAGE and analyzed by Coomassie blue staining (top) and Western blotting (middle and bottom) using a phospho-threonine specific antibody. In the bottom panel the PVDF membrane was treated with 10 units alkaline phosphatase in FastAP buffer (ThermoFisher Scientific) for 60 minutes at 37°C before the α-phospho-threonine antibody was applied.

To explain the surprising results of the *in vivo* phosphorylation assays, we reasoned that QseF is phosphorylated at a second site, masking its phosphorylation at Asp56. We speculated that the additional phosphorylation(s) may take place in the QseF C-terminus comprising the σ^54^ interaction domain and the DNA-binding domain (subsequently designated as QseF-CTD). Phosphorylation of response regulators outside their receiver domains has been observed in several cases [38]. Therefore, we split the protein and expressed the QseF N-terminus comprising the receiver domain (subsequently designated as QseF-NTD) and the QseF-CTD separately, both provided with C-terminal Strep-tags for subsequent pull-down and detection (Fig 5A). In addition, a QseF-NTD variant was generated carrying the D56A exchange of the phosphorylation site. Bacteria producing the various QseF-Strep variants from plasmids (Fig 5B, left panel) were labeled with [^32^P] followed by pull-down of the QseF variants, which was confirmed by Western blotting (Fig 5B, middle panel). Indeed, autoradiography detected phosphorylation signals for both, the QseF-NTD and the QseF-CTD (Fig 5B, right panel). Importantly, no phosphorylation of the QseF-NTD carrying the D56A substitution was observable (Fig 5B, right panel). These data show that D56 is the single site phosphorylated in the QseF receiver domain, whereas the additional phosphorylation signal localizes in the QseF-CTD. Western blot analysis of purified proteins using an antiserum specific for phospho-tyrosine residues generated no signals. However, a phospho-threonine specific antiserum detected full-length QseF and the QseF-CTD (Fig 5C, top and middle panel). In contrast, the QseF-NTD and the response regulator PhoB, which was included as a control, were not detectable (Fig 5C, middle panel, lanes 1 and 4). Treatment of the PVDF membrane with alkaline phosphatase prior to application of the antiserum erased the signals for full-length QseF and the QseF-CTD (Fig 5C, bottom panel). Thus, QseF is phosphorylated at D56 in the receiver domain and presumably at unknown threonine or serine residue(s) in the CTD.

### QseG triggers phosphorylation of the QseF receiver domain by kinase QseE

**Fig 6:**
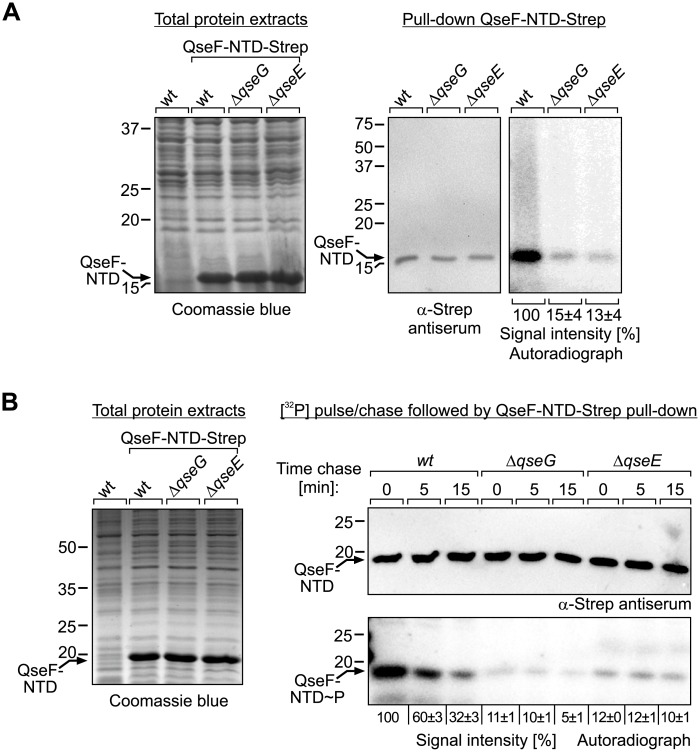
QseG and QseE are required for phosphorylation of the QseF receiver domain. **A**. StrepTactin pull-down of QseF-NTD following metabolic [^32^P] labeling. Strains Z197 (*wild-type*), Z477 (Δ*qseG*) and Z970 (Δ*qseE*) harboring plasmid pYG279 (encoding QseF-NTD-Strep) were grown in LB and synthesis of QseF-NTD-Strep following addition of IPTG was verified by SDS-PAGE of total protein extracts (left panel). Cells were labelled using [^32^P] and QseF-NTD-Strep was subsequently isolated by pull-down and separated by 15% SDS-PAGE. Gels were analyzed by Western blotting (middle panel) using an antibody directed against the Strep-tag and autoradiography (right panel). **B**. Pulse-chase experiment to assess QseE phosphatase activity *in vivo*. The transformants used in (A) were labelled using [^32^P] and subsequently chased with “cold” phosphorus for the indicated times. Synthesis of QseF-NTD-Strep was confirmed by SDS-PAGE of total protein extracts (left panel). Following chase, the QseF-NTD-Strep was pulled down and analyzed by Western blotting (top panel, right) and autoradiography (bottom panel, right). Obtained phosphorylation signals were quantified and quantifications are displayed below the autoradiographs. Phosphorylation signal intensities are expressed in percentage of the signal obtained in the *wild-type* following pulse-labeling (no chase).

Next, we used the QseF-NTD construct to clarify the question whether QseE and QseG are required for phosphorylation of QseF at the D56 residue in the receiver domain. To this end, the plasmid encoding the C-terminally Strep-tagged QseF-NTD was introduced in isogenic *wild-type*, *ΔqseG* and *ΔqseE* strains. Once again, the bacteria were grown in LB supplemented with IPTG to induce synthesis of recombinant proteins (Fig 6A, left panel) and subsequently labelled with [^32^P] phosphoric acid. Finally, the QseF-NTD was isolated by pull-down and eluates were analyzed by Western blotting using anti-Strep antiserum and by autoradiography. Western blotting proved successful isolation of the QseF-NTD from all three strains (Fig 6A, middle panel). The autoradiograph revealed a strong phosphorylation signal for the QseF-NTD isolated from the *wild-type* strain. In contrast, 7- and 8-fold reduced phosphorylation signal intensities were obtained, when the QseF-NTD was isolated from the *ΔqseG* and *ΔqseE* mutants (Fig 6A, right panel). These data show that both QseE and QseG are required for efficient phosphorylation of the QseF receiver domain. As QseG is unable to increase phosphorylation of QseF in the *qseE* mutant (Fig 6A), it can be concluded that QseG acts through QseE to stimulate phosphorylation of QseF and thereby *glmY* expression.

Some histidine kinases are bi-functional and exhibit in addition to phosphotransferase also phosphatase activity towards the cognate response regulator [1]. Kinase activity of QseE towards QseF was demonstrated previously [39], but whether QseE has also phosphatase activity is unknown. In principle, QseG could increase phosphorylation of QseF either by stimulating phosphotransferase activity or by inhibiting phosphatase activity of QseE. To gain initial insight into how QseG governs phosphorylation of the QseF receiver domain, we carried out [^32^P] pulse-chase experiments to follow the fate of the QseF-D56 phosphorylation signal in a time course. Therefore, we once again labelled the *wild-type*, *ΔqseG* and *ΔqseE* strains producing the Strep-tagged QseF-NTD (pulse), but subsequently stopped further incorporation of the [^32^P] label by addition of “cold” phosphorus (chase). Samples were harvested at 0, 5 and 15 min following chase and the QseF-NTD was subsequently isolated by StrepTactin pull-down and analyzed as before (Fig 6B). The phosphorylation signal for the QseF-NTD rapidly diminished within 15 min in the wild-type strain, whereas such a decrease was not observable in the *ΔqseE* mutant (Fig 6B, compare lanes 1–3 with 7–9). This result indicates that QseE possesses phosphatase activity and is responsible for dephosphorylation of the QseF receiver domain in the wild-type strain. In case QseG would act by inhibition of QseE phosphatase activity, an accelerated dephosphorylation of the QseF-NTD is expected in the *ΔqseG* mutant as compared to the wild-type. However, this was not the case: The QseF-NTD phosphorylation signal also decreased over time in the *ΔqseG* mutant, but not faster than in the wild-type strain (Fig 6B, lanes 4–6). In conclusion, QseG appears not to act by inhibition of phosphatase activity, suggesting that it increases phosphorylation of the QseF receiver domain through stimulation of QseE phosphotransferase activity.

### Outer membrane lipoprotein QseG and kinase QseE physically interact in the periplasm

**Fig 7:**
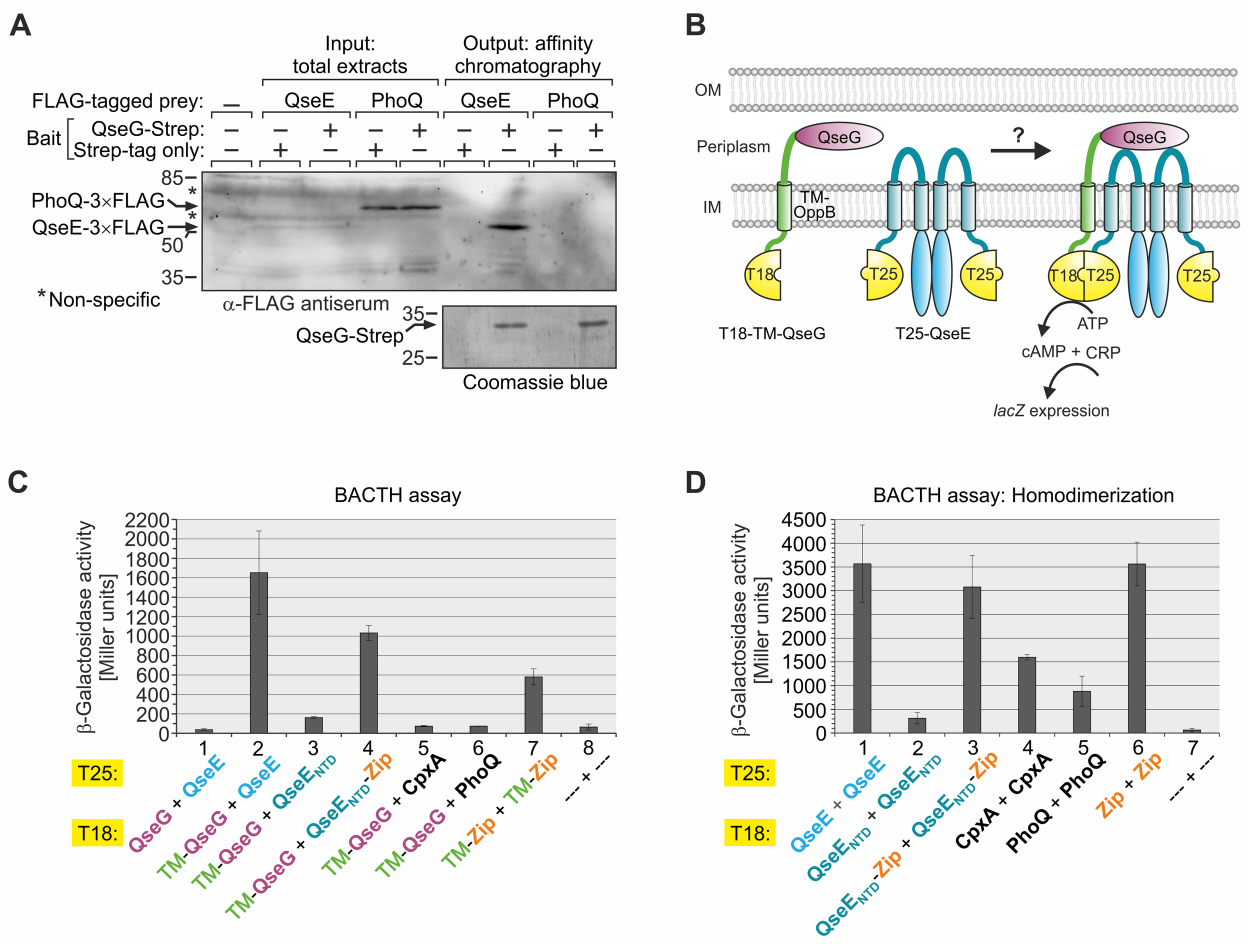
QseG and QseE interact in the periplasm. **A**. Pull-down assay based on StrepTactin affinity chromatography assessing interaction of QseG and QseE. Strains Z952 and Z986 were employed, which carry *qseE*-3xFLAG and *phoQ*-3xFLAG alleles on the chromosome, respectively. These strains either harbored plasmid pYG191 carrying the *qseG-strep* allele under *P*_*tac*_ control or the isogenic plasmid pBGG237 encoding the Strep peptide only. Following induction of *P*_*tac*_-controlled genes by IPTG, lysates were prepared (left, “input”) and subjected to StrepTactin affinity chromatography. Presence of QseG-Strep in the elution fractions was verified by SDS-PAGE/Coomassie blue staining (bottom panel, right). Lysates (“input”) and elution fractions (“output”) were separated alongside by SDS-PAGE and analyzed by Western blotting using α-FLAG antiserum (top panel). Strain Z197 lacking the FLAG epitope served as negative control (lane 1). Non-specific signals are indicated with asterisks. **B**. Cartoon illustrating usage of modified BACTH [46] to monitor interaction of QseG with QseE. QseG lacking the N-terminal signal sequence (Δ aa 1–25) was fused to the C-terminus of the TM of OppB for periplasmatic localization, while the N-terminus of the OppB TM is fused to the cytoplasmic CyaA-T18 fragment. The N-terminus of QseE was fused to the T25 fragment of CyaA. In case of interaction between periplasmic QseG and the periplasmic loop of QseE, the cytosolic T18 and T25 fragments are in proximity to reconstitute CyaA activity. Production of cAMP leads to elevated *lacZ* expression, which was monitored. **C**. BACTH assay assessing interaction of QseG with QseE. The following plasmid combinations were tested in reporter strain BTH101 (left to right): pYG196/pYG199, pYG242/pYG199, pYG242/pYG259, pYG242/pYG255, pYG242/pYG248, pYG242/pYG250, pUT18C-TM-zip/ pKT25-TM-zip, pUT18C/pKT25. **D**. BACTH analyses addressing homo-dimerization of QseE, CpxA and PhoQ. The following plasmid combinations were introduced into BTH101 and tested (left to right): pYG246/pYG199, pYG256/pYG259, pYG257/pYG255, pVK2/pYG248, pVK1/pYG250, pUT18C-zip/pKT25-zip (positive control), pUT18C/pKT25 (negative control). β-galactosidase activities were determined from cells grown to stationary phase.

QseG faces the periplasmic leaflet of the outer membrane [24, 31]. On the other hand, kinase QseE contains a helical periplasmic domain of 140 amino acids between its two transmembrane domains (TMs; [40]). This topological arrangement makes a physical interaction of QseG with the periplasmic domain of QseE feasible. Physical interaction of outer membrane lipoproteins with the periplasmic domains of cytoplasmic membrane proteins has been demonstrated in several cases [41–43]. To investigate whether QseG and QseE interact, we used a ligand fishing approach based on StrepTactin affinity chromatography, which allows for pull-down of membrane proteins by cytoplasmic or periplasmic interaction partners as demonstrated previously [4, 44]. For detection of the prey protein QseE, the sequence encoding the 3×FLAG epitope was fused in frame to the 3’ end of *qseE* encoded at its natural chromosomal locus. An isogenic strain carrying the 3×FLAG epitope sequence fused to the 3’ end of *phoQ* served as negative control. Similar to QseE, PhoQ is a histidine kinase that possesses two N-terminal TMs encompassing a large domain extruding into the periplasm. QseG carrying a C-terminal Strep-tag was used as bait and produced from a plasmid in the latter two strains. A complementation assay confirmed functionality of the QseG-Strep protein (S8 Fig). The same strains, but producing solely the Strep-peptide rather than QseG-Strep served as negative controls. Analysis of total cell extracts by Western blotting revealed a clear signal for the PhoQ-3×FLAG protein (MW = 58.12 kDa) in addition to several non-specific bands, whereas only a faint band for QseE-3×FLAG (MW = 56.15 kDa) was detectable (Fig 7A, “input”), reflecting the notoriously weak expression level of *qseE* (see above and [9]). The various strains were subjected to the StrepTactin affinity purification protocol and inspection of the eluates proved successful purification of QseG-Strep (Fig 7A, “output” bottom panel). Western blotting analysis of the eluates revealed a strong enrichment of QseE-3×FLAG when QseG-Strep was used as bait, whereas no signals were obtained when the Strep-peptide was produced or when PhoQ-3×FLAG was assessed as potential prey, providing proof of specificity of the QseE-QseG interaction detected by this approach (Fig 7A).

To further characterize interaction of QseG and QseE, we used the bacterial adenylate-cyclase based two-hybrid system (BACTH), which relies on interaction-mediated reconstitution of adenylate cyclase activity in *E*. *coli* [45]. In BACTH, the complementary T18- and T25-fragments of *Bordetella pertussis* adenylate cyclase are assembled to a functional enzyme through interaction of candidate proteins that are fused to these fragments. The classical BACTH is restricted to interactions within the cytoplasm or the cytoplasmic membrane, but more recently modified BACTH vectors have been developed, which allow to assess extra-cytoplasmic protein interactions [46]. In this case, a membrane domain of the *E*. *coli* OppB protein is inserted in the fusion protein between the CyaA-fragment and the candidate protein, resulting in extrusion of the latter into the periplasm, while the N-terminal CyaA fragment stays in the cytoplasm. Therefore, we fused the sequence encoding QseG (but lacking the first 25 codons encoding the N-terminal export signal) to the 3’ end of the *T18-TM*_*oppB*_ fusion gene (Fig 7B). Of note, deletion of the export signal in the context of the *wild-type* QseG protein rendered the protein inactive, supporting the idea that QseG must leave the cytoplasm to stimulate *glmY* expression (S9 Fig, columns 1–4). The T18-TM_oppB_-QseG BACTH fusion construct was then tested for interaction with QseE, which was fused to the C-terminus of the T25 fragment (Fig 7B). Indeed, β-galactosidase assays reflecting cAMP synthesis detected activity fairly above the level of the negative control, in which the unfused CyaA fragments were addressed (Fig 7C, columns 2 and 8). Activity even exceeded the positive control, which detects homodimerization of the leucine zipper of the yeast transcription factor Gcn4 in the periplasm (Fig 7C, compare columns 2 and 7). No interaction was detectable when QseG [Δ aa 1–25] was directly fused to the T18 fragment omitting the TM_oppB_ domain in the fusion protein (Fig 7C, column 1), confirming that QseG must leave the cytoplasm to interact with QseE. To provide further proof of specificity of the detected QseG-QseE interaction, we also tested interaction of the T18-TM_oppB_-QseG fusion with membrane-bound histidine kinases CpxA and PhoQ, which exhibit similar membrane-topologies as QseE, i.e. they possess large periplasmic domains encompassed by two N-terminally located TMs. However, only back-ground activities could be measured in these cases (Fig 7C, columns 5 and 6). BACTH assays addressing homodimerization of the kinases proved functionality of the fusion proteins (Fig 7D, columns 1, 4, 5).

**Fig 8:**
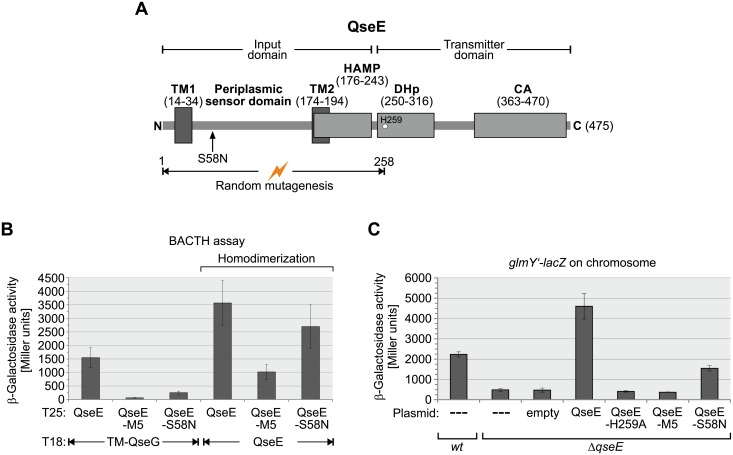
Mutations in the QseE N-terminus impairing interaction with QseG concomitantly decrease QseE activity. **A**. Schematic representation of the domain architecture of sensor kinase QseE. Amino acid residues encompassing the respective domains are given in parenthesis and the phosphorylated histidine residue H259 is depicted by a circle. Positions are according to the EcoCyc database [6]. The HAMP domain has been predicted by Pfam [68]. The sequence coding for amino acid residues 1–258 was randomly mutagenized and the resulting QseE mutant library was phenotypically screened for loss of interaction with QseG in the context of BACTH. The position of the thereby identified S58N substitution is indicated with an arrow. **B**. Quantitative BACTH analysis of the interaction potential of T25-QseE variants identified in the screen for loss of interaction with T18-TM-QseG. The following plasmid combinations were tested in reporter strain BTH101 (left to right): pYG242/pYG199; pYG242/pYG199_TM1; pYG242/pYG199_1.6; pYG246/pYG199; pYG246/pYG199_TM1; pYG246/pYG199_1.6. **C**. Complementation analysis assessing the ability of QseE variants to activate transcription from promoter *P*_*glmY*_. The following plasmids encoding the proteins under *P*_*tac*_ control were introduced into the Δ*qseE* mutant Z970 carrying a *glmY’-lacZ* reporter fusion and the β-galactosidase activities were determined: pKESK23 (empty vector, column 3), pYG221 (wt-QseE, column 4), pYG221-H259A (QseE-H259A, column 5), pYG221-TM1 (QseE-M5, column 6), pYG221-S58N (QseE-S58N, column 7). As controls, un-transformed *wild-type* (Z197) and Δ*qseE* (Z970) strains were used (first two columns).

Next, we wanted to confirm that QseG interacts with the N-terminus of QseE comprising the periplasmic loop. Therefore, we tested interaction of the QseG fusion protein with the N-terminus of QseE (residues 1–250; subsequently designated QseE_NTD_) lacking the C-terminal transmitter domain (Fig 8A). However, in this case only background activities were detectable (Fig 7C, columns 3 and 8). Dimerization of histidine kinases is usually mediated through the transmitter domains [1] and accordingly homodimerization of the QseE_NTD_ was greatly impaired (Fig 7D, columns 1 and 2). To test, whether the loss of interaction with QseG resulted from the inability of QseE_NTD_ to form dimers, we fused the leucine zipper homodimerization domain of Gcn4 to the C-terminus of QseE_NTD_. Indeed, this procedure rescued dimerization (Fig 7D, column 3) and also partially restored interaction with QseG (Fig 7C, column 4). Taken together, the data indicate that QseG binds the N-terminus of QseE in the periplasm and that dimerization of QseE is a prerequisite for this interaction.

### A mutation in the QseE periplasmic domain impairs interaction with QseG and concomitantly decreases kinase activity

Our data suggested that activation of QseE by QseG may require their physical interaction in the periplasm. To obtain insight, we searched for mutations in the QseE N-terminus decreasing interaction with QseG. To this end, we randomly mutagenized the sequence encoding the QseE N-terminus (aa 1–258) within the full-length *T25-qseE* construct by error prone PCR and screened the resulting library of QseE mutants in context of BACTH for variants showing decreased interaction with QseG (Fig 8A). In addition to mutants carrying stop- or frameshift mutations, which were not further analyzed, two mutants carrying exclusively amino acid exchanges were isolated. One mutant (subsequently designated “QseG-M5) carried five exchanges (i.e. F19L, L21H, I22R, L23P, L24P) in TM1, while the other mutant received a single amino acid exchange (S58N) in the periplasmic loop (Fig 8A and S10 Fig). Quantitative assays revealed that interaction of QseE with QseG is abrogated by the M5 mutation and significantly decreased when the S58N exchange was present (Fig 7B, columns 1–3). A pull-down assay using QseG-Strep as bait confirmed the decreased interaction potential of the QseE-S58N variant (S11 Fig). In this case, presence of the S58N mutation reduced the amount of co-purifying QseE-3×FLAG protein ~3-fold (S11 Fig, compare lanes 7 and 9). Thus, interaction of QseE with QseG is impaired by the S58N mutation but not completely abolished. To test for kinase activity, the various QseE variants were placed on plasmids under *P*_*tac*_-promoter control and used to complement a *ΔqseE* mutant strain carrying the *glmY’-lac*Z reporter fusion on the chromosome (Fig 8C). Indeed, the M5 mutation abolished QseE activity as judged from comparison with the empty vector control and an inactive QseE-H259A mutant carrying a substitution in the QseE autophosphorylation site (Fig 8C, compare columns 1–6). However, as indicated by BACTH, the QseE-M5 mutant was also strongly impaired in homodimerization (Fig 8B, columns 4 and 5). Therefore, the mutations in TM1 might interfere with proper membrane insertion of QseE rather than to specifically abrogate interaction with QseG. In contrast, the QseE-S58N mutant was not significantly impaired in homodimerization (Fig 8B, columns 4 and 6). Strikingly, the QseE-S58N mutant showed a 3-fold decreased potential to activate transcription of *glmY* as compared to *wild-type* QseE (Fig 8C, compare columns 4 and 7). The residual activation potential of QseE-S58N is still dependent on QseG (S12 Fig). Thus, the S58N exchange diminishes interaction with QseG and concomitantly lowers activity of QseE. This result supports a model in which QseG activates QseE kinase activity through interaction.

### Epinephrine stimulates QseE phosphorylation and thereby *glmY* transcription in a QseG-dependent manner in the stationary growth phase

**Fig 9:**
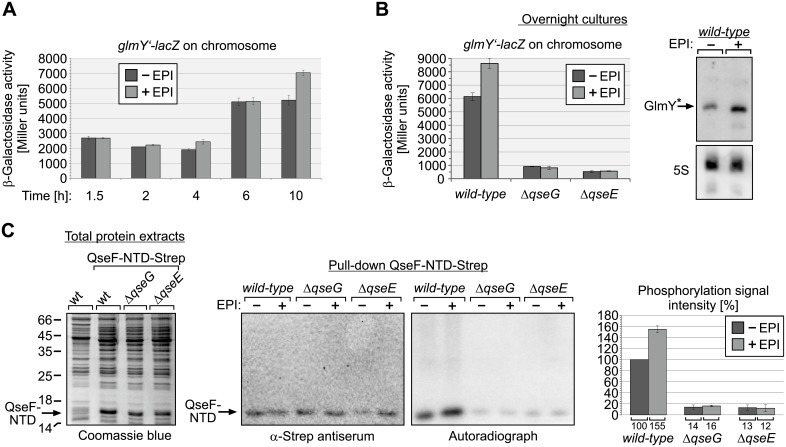
Impact of epinephrine on the QseE/QseF TCS. **A**. Transcription of a chromosomal *glmY’-lacZ* fusion in *wild-type* strain Z197 during growth in absence and presence of 150 μM epinephrine (EPI). Cells were inoculated in LB with or without EPI to an OD_600_ = 0.1. Following the indicated times of incubation, samples were harvested and the β-galactosidase activity was determined. **B**. Expression of *glmY* in *wild-type* (Z197), Δ*qseG* (Z477) and Δ*qseE* (Z970) strains following 16 h of growth overnight in absence and presence of 150 μM Epi. Subsequently, the β-galactosidase activities were determined to assess expression of the *glmY’-lacZ* fusion carried on the chromosomes of these strains. In addition, total RNA was extracted from the *wild-type* strain and GlmY amounts were analyzed by Northern Blotting (right panel, top). Detection of 5S rRNA served as loading control (right panel, bottom). **C**. Effect of epinephrine on phosphorylation of the QseF receiver domain *in vivo*. Strains Z197 (*wild-type*), Z477 (Δ*qseG*) and Z970 (Δ*qseE*) harboring plasmid pYG279 (encoding QseF-NTD-Strep) were grown in the presence of IPTG for induction of QseF-NTD-Strep and subsequently subjected to metabolic ^32^P labeling and StrepTactin pull-down for isolation of QseF-NTD. Where indicated 150 μM Epi was added to the cells prior to addition of H_3_[^32^P]O_4_. Proper synthesis of QseF-NTD-Strep was confirmed before labelling by analysis of total protein extracts by SDS-PAGE and Coomassie blue staing (left panel). Pull-down fractions were analyzed by Western blotting using α-Strep antiserum for successful isolation of the QseF-NTD-Strep (middle panel) and by autoradiography (right panel). Obtained phosphorylation signals were quantified from at least three independent experiments and quantifications are displayed in the diagram (right). Phosphorylation signal intensities are expressed in percentage of the signal obtained in the *wild-type* in the absence of EPI.

A previous study reported that QseE of EHEC responds to epinephrine *in vitro* by increased autophosphorylation [31]. To learn whether epinephrine has also a role for QseE activity in *E*. *coli* K-12, we studied the impact of epinephrine on *glmY* transcription during growth. Epinephrine did not change *glmY* transcription during the exponential and early stationary growth phase (Fig 9A). However, after 10 h growth in presence of epinephrine a somewhat higher *glmY* transcription level became evident in the epinephrine treated culture (Fig 9A). To confirm this result, we determined *glmY* transcription levels in overnight cultures incubated for ~16h in epinephrine containing LB medium. Once again, higher *glmY’-lacZ* levels were observable in the *wild-type* strain in presence of epinephrine and Northern blot analysis confirmed that GlmY accumulated to higher amounts in this case (Fig 9B). In contrast, the *ΔqseG* and *ΔqseE* mutant strains showed only low *glmY* transcription levels and failed to respond to epinephrine (Fig 9B). These results suggested that epinephrine might stimulate QseE autophosphorylation in a QseG-dependent manner. To address this possibility, we studied phosphorylation of the QseF receiver domain by metabolic [^32^P] labelling followed by pull-down *in vivo*. The Strep-tagged QseF-NTD was overproduced from a plasmid in *wild-type*, *ΔqseG* and *ΔqseE* strains (Fig 9C, left) and subsequently cells grown to stationary phase were labelled in the absence and presence of epinephrine and the QseF-NTD was isolated by pull-down using StrepTactin coated magnetic beads (Fig 9C, middle panel). Indeed, epinephrine moderately stimulated phosphorylation of the QseF-NTD in the *wild-type* strain (Fig 9C, right panel and diagram). In the *ΔqseG* and *ΔqseE* mutants, however, phosphorylation of the QseF-NTD was strongly decreased as observed before (Fig 6) and epinephrine had no effect on the remaining phosphorylation signal (Fig 9C, right panel and diagram). Thus, epinephrine is capable to stimulate QseE phosphorylation even in *E*. *coli* K-12, but this effect requires QseG and solely occurs in the stationary growth phase.

## Discussion

**Fig 10:**
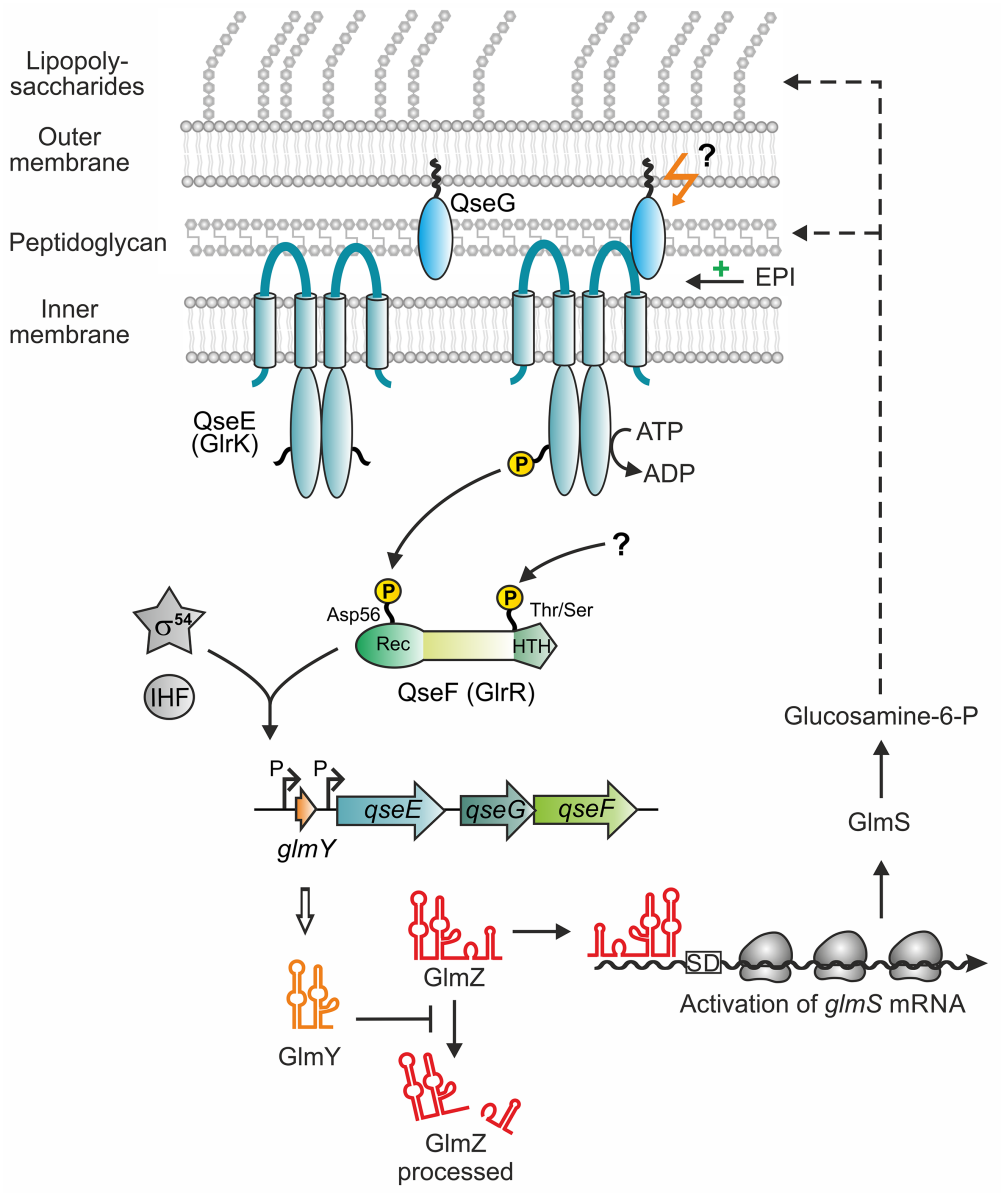
Model for control of sRNA *glmY* transcription by the QseE/QseG/QseF three-component system in *E*. *coli* K-12. The model summarizes data obtained in the current and in previous studies [8, 9, 16]. In the absence of QseG, kinase QseE is inactive and unable to activate response regulator QseF. QseG is a lipoprotein attached to the outer membrane and binds the periplasmic loop of kinase QseE. Interaction with QseG may activate kinase QseE to phosphorylate response regulator QseF at residue Asp56 in the receiver domain. Whether this interaction occurs with membrane-attached or soluble QseG remains unclear. The host hormone epinephrine moderately stimulates phosphorylation of QseF by QseE in a QseG-dependent manner when cells reside in the stationary growth phase. In addition, QseF is phosphorylated by an unknown activity in the C-terminus, presumably at Thr or Ser residue(s). Assisted by the integration host factor IHF, phosphorylated QseF binds to conserved sites upstream of *glmY* and activates *glmY* transcription from a σ^54^-dependent promoter. The sRNA GlmY in turn counteracts degradation of the homologous sRNA GlmZ through sequestration of protein RapZ, which is required for GlmZ decay. Through a base-pairing mechanism GlmZ activates synthesis of glucosamine-6-phosphate synthase, which generates glucosamine-6-phosphate—the first dedicated metabolite for synthesis of peptidoglycan and lipopolysaccharides.

In this work, we show that the outer membrane lipoprotein QseG is an indispensable component of the QseE/QseF TCS, reflecting the conserved co-localization of the *qseEGF* genes in one operon. Genetic and *in vivo* phosphorylation studies indicate that QseG triggers phosphorylation of QseE/QseF *in vivo*, constituting a “three-component system” (Figs 1, 2 and 6). QseG binds the large periplasmic domain of kinase QseE (Fig 7) and this interaction likely triggers QseE autophosphorylation activity or stimulates QseE/QseF phosphoryl-group transfer (Fig 10). Such a model is supported by identification of the S58N exchange located in a conserved region in the periplasmic domain of QseE (S10 Fig), which impairs interaction with QseG and concomitantly decreases activity of QseE/QseF (Fig 8; S11 Fig). The data are consistent with a model, in which the outer membrane protein QseG activates kinase QseE by interaction thereby increasing the level of phosphorylated QseF, which in turn activates the σ^54^-dependent promoters upstream of *glmY* and *rpoE*, both encoding central regulators of cell envelope homoeostasis (Fig 10).

In agreement with our results, QseG was shown also to be required for activation of the σ^54^-dependent *rpoE* promoter by response regulator QseF [10]. In this case, QseG was identified in a screen as a multi-copy activator of the *rpoE* σ^54^-promoter. QseG carries a 25 aa long export sequence at the N-terminus, including a so-called “lipobox”, and does not contain a Lol avoidance motif (S13 Fig). Therefore, it is exported to the periplasm (S1 Fig) and predicted to attach to the inner leaflet of the outer membrane via lipidation of residue Cys26, which should become the new N-terminal amino acid following cleavage of the signal peptide [47]. Consistently, EHEC QseG, which is identical with QseG from *E*. *coli* K-12 (S13 Fig), was shown to localize to the outer membrane, but being inaccessible to proteinase K digestion from the exterior [24, 31]. This extracytoplasmic localization is in perfect agreement with our observation that QseG must leave the cytoplasm in order to interact with QseE and to activate *glmY* transcription (Fig 7C and S9 Fig). However, it should be stressed that the exact localization of QseG within the periplasmic compartment is apparently not crucial for its activity. Mutation of the presumably lipidated Cys26 residue has only a moderate effect on QseG activity and QseG even retains significant activity when carrying a V27D Lol avoidance motif (S9 Fig) leading to its retention in the cytoplasmic membrane [47]. Obviously, QseG can reach and bind QseE regardless of its specific localization within the periplasmic space. In any case, the 237 amino acids long QseG protein is sufficiently large to form a trans-envelope complex with QseE [42].

The architecture of the QseE/QseG/QseF three-component system is remarkably reminiscent of the Cpx and Rcs envelope stress response systems, which are also built around two-component systems that employ outer membrane lipoproteins for signal perception and activation of the phosphorylation cascade [48]. Under normal conditions, the lipoprotein RcsF is threaded into β-barrel Omp proteins and thereby sequestered at the outer membrane [41, 49]. Stress prevents incorporation of RcsF into these complexes leading to accumulation of RcsF remaining exposed in the periplasm. This enables the outer-membrane attached RcsF to interact with the inner membrane protein IgaA, thereby releasing the Rcs phospho-relay system from IgaA-mediated inhibition [41, 42]. In the Cpx system, the outer membrane lipoprotein NlpE activates the CpxA/CpxR TCS, presumably through direct interaction with kinase CpxA or its periplasmic inhibitor CpxP [50]. As a common principle, phosphorylation of the Rcs and Cpx systems is triggered by availability of the cognate lipoproteins for interaction in the periplasm. Accordingly, both systems can be activated by artificially increasing the levels of these lipoproteins [51, 52]. A similar scenario is observed here, as activity of the QseE/QseF TCS directly correlates with *qseG* expression levels (Fig 2 and S5 Fig). Apparently, QseE/QseF phosphorylation activity is limited by availability of QseG in the periplasm. It is tempting to speculate that cells control the levels of “free” QseG available for interaction with QseE, to adjust QseE/QseF activity accordingly. Interestingly, in EHEC QseG was recently found to interact with the LEE-encoded protein SepL, which serves as gate-protein for the type III secretion system used to translocate effector proteins into host cells [24]. Albeit the role of this interaction remained unclear, it could serve to sequester QseG making it unavailable for interaction with QseE. Such a mechanism could fine-tune synthesis of type III secretion system components, as their expression is controlled by QseE/QseF through GlmY/GlmZ [29]. However, SepL is absent in *E*. *coli* K-12 indicating that interaction of QseG with QseE must be differently controlled, which will be the subject of future studies.

Moreover, we show that QseF also responds to Epi in *E*. *coli* K-12, but moderately and exclusively in the stationary growth phase. Under these conditions Epi increases QseF phosphorylation and concomitantly *glmY* transcription 1.5-fold and this effect requires both, kinase QseE and QseG (Fig 9). In respect to its limited impact, it appears that Epi is not a major stimulus for the QseE/QseF TCS in *E*. *coli* K-12, which apparently is, at least partially, already in the “on-state” under standard laboratory conditions (Figs 1, 2 and 6). As QseE requires QseG to respond to Epi (Fig 9), it appears debatable whether QseE is able to sense Epi on its own [31]. Interaction of Epi with the QseE periplasmic domain could also not be observed by NMR [40]. Therefore, interaction of QseG with epinephrine appears to be a possible mechanism. However, we cannot exclude that the weak stimulatory effect of Epi on QseE/QseF phosphorylation is indirect, and may perhaps involve a putative interaction partner of QseG or even epinephrine degradation products. EHEC QseE was also reported to sense phosphate and sulfate ions as it responds with increased autophosphorylation to these signals *in vitro* [31]. However, when tested in a minimal medium, phosphate and sulfate had no impact on *glmY* transcription in *E*. *coli* K-12 (S14 Fig). Apparently, through employment of QseG, QseE senses different cues in *E*. *coli* K-12, most likely signal(s) derived from the cell envelope. In agreement, activity of QseF was observed to increase in a *waaC* mutant [10]. Gene *waaC* encodes LPS heptosyltransferase I and its absence causes defects in LPS biosynthesis.

In addition, there is also no Epi-dependent cross-phosphorylation of QseF by histidine kinase QseC in *E*. *coli* K-12, as Epi is unable to increase phosphorylation of QseF in the absence of QseE (Fig 9C). This is in contrast to EHEC, in which QseC was reported to contribute to QseF phosphorylation as it is able to cross-phosphorylate QseF *in vitro* [32]. In EHEC, the QseB/QseC TCS was also described to cross-talk with the QseE/QseF TCS at the level of *glmY* transcription: In addition to QseF, also response regulator QseB was shown to bind to the EHEC *glmY* promoter region, thereby stimulating *glmY* expression two-fold [29]. However, in *E*. *coli* K-12 deletion of *qseB* or *qseC* has no effect on *glmY* transcription (S15 and S16 Figs), which might be explained by differences in the sequences of the predicted QseB binding sites [29]. Therefore, the activities of the QseB/QseC and QseE/QseF TCSs appear to be well separated in *E*. *coli* K-12. It even appears unlikely that QseF can receive phosphoryl-groups from any other histidine kinase than QseE *in vivo*, as *glmY* transcription from the σ^54^-promoter is abolished in a mutant lacking kinase QseE (Figs 2 and 8C; [9]). The weak phosphorylation of the QseF receiver domain remaining detectable in the absence of QseE or QseG (Figs 6 and 9C) might result from non-physiological cross-talk as we had to overproduce the QseF-NTD in these experiments, potentially providing a sink for non-cognate phosphorylations. Alternatively, these cross-phosphorylations may not be robust as they could be removed through QseE phosphatase activity in *wild-type* cells (Fig 6B). Phosphatase activities of histidine kinases were shown to be crucial to prevent aberrant phosphorylations of response regulators by non-cognate kinases [53].

In this work, we studied phosphorylation of QseF *in vivo* using metabolic [^32^P] labelling, which is a method usually not considered in TCS research [54], albeit it allows to snapshot protein phosphorylation states in the living cell [55]. Using this approach, we also detected an additional phosphorylation signal for the QseF-CTD, suggesting that QseF is at least doubly phosphorylated (Figs 4 and 5). The C-terminal phosphorylation was also detectable by an antiserum recognizing phosphorylated Thr- and Ser-residues (Fig 5C). Meanwhile several response regulators are known to become additionally phosphorylated by Ser/Thr kinases interfering with their function [38, 56]. In *E*. *coli*, two serine/threonine kinases, SrkA (a.k.a YihE) and YeaG, have been characterized [57, 58], but they are not required for phosphorylation of the QseF-CTD (S17 Fig). The source and role of this additional phosphorylation signal must be addressed in future research.

## Materials and methods

### Strains, plasmids and growth conditions

*E*. *coli* strains were routinely grown in Lysogeny broth (LB medium) at 37°C or in case of bacterial two hybrid assays at 28°C. When required, antibiotics were added to the following concentrations: ampicillin (100 μg/ml), kanamycin (30 μg/ml), spectinomycin (75 μg/ml) and chloramphenicol (15 μg/ml). *E*. *coli* strains and plasmids used in this study are described in S1 and S2 Tables and oligonucleotides are listed in S3 Table under “Supporting information”. Details on plasmid constructions are described in S1 Text under “Supporting information”. Deletions in the chromosomal *qseEGF* operon were constructed by λ red recombination using plasmid pKD3 as template as described and the oligonucleotides specified in S1 and S3 Tables [59]. FLAG-tagging of chromosomal genes was performed as described previously [60] using oligonucleotides BG1305/BG1306 for *qseG*, BG902/BG903 for *phoQ*, BG968/BG969 for *qseE* and plasmid pSUB11 as template. Ectopic integration of *glmY’-lacZ* reporter gene fusions into the *λattB* site on the *E*. *coli* chromosome was achieved as described before [8, 61]. Established alleles were moved between strains by general transduction using *E*. *coli* phage T4GT7 [62]. Strains were cured from resistance gene cassettes using FLP recombinase encoded on plasmid pCP20 as described [59].

### Isolation of cell envelope proteins

Cell envelope fractions containing soluble periplasmic and outer membrane proteins were isolated as described [34]. Briefly, *E*. *coli* strain Z197 harboring either plasmid pYG191 coding for QseG-Strep or the isogenic plasmid pBGG237 encoding only the Strep-peptide was grown in 100 ml M9 minimal medium supplemented with 1% maltose, 0.1% casamino acids, thiamine (1 μg/ml) and L-proline (40 μg/ml). One half of each culture was harvested in the exponential growth phase (OD_600_ ~0.5), whereas the remaining half was harvested in the stationary growth phase. Cells were pelleted by centrifugation, gently re-suspended in 200 μl TSE buffer (200 mM Tris-HCl pH 8.0, 500 mM sucrose, 1 mM EDTA) and incubated on ice for 60 min. The TSE-soluble proteins were separated from the insoluble fractions by centrifugation (16000 g, 4°C, 45 min) and 6.25 μg of the supernatants containing the periplasmic extracts were analyzed by SDS PAGE and Western blotting, respectively.

### RNA extraction and Northern blotting

RNA extraction and Northern blotting was performed as described before [19]. Bacteria were grown in LB for the indicated times and cells were harvested by centrifugation (2 min, 4°C, 11000 rpm) and frozen in liquid nitrogen. RNA was extracted using the RNeasy mini kit (Qiagen) according to the manufacturer’s instructions. Digoxigenin-labeled RNA probes against GlmY and 5S RNA were obtained by *in vitro* transcription using the DIG-Labelling kit (Roche Diagnostics) and specific PCR fragments as templates. Primer pairs used for PCR were BG260/BG261 for *glmY* and BG287/BG288 for *rrfD* (5S). T7 RNA polymerase promoter sequences were introduced during PCR by incorporation of the reverse primer. 2.5 μg of total RNA/lane were separated on a 7 M urea/TBE/8% polyacrylamide gel and subsequently transferred to a positively charged nylon membrane (GE Healthcare) by electroblotting in 0.5×TBE at 15 V for 1 h. Probe hybridization and detection were performed according to the supplier’s instruction (DIG RNA Labelling kit, Roche Diagnostics).

### Determination of β-galactosidase activity

β-Galactosidase activity assays were performed as described previously [63]. Activities were determined from exponentially growing cells (OD_600_ = 0.5–0.8) if not otherwise indicated. Reported values are the average of at least three measurements using independent cultures.

### Labeling of phosphorylated proteins by [^32^P] *in vivo*

Metabolic labeling of phosphorylated proteins using H_3_[^32^P]PO_4_ was carried out as described previously with slight modifications [64, 65]. Briefly, bacteria were grown in LB medium to late exponential phase (OD_600_ ~ 0.5–0.8) and subsequently expression of plasmid-encoded proteins was induced using 1 mM IPTG. Following an additional incubation for 30 min, cells were washed and further incubated for 1 h in phosphate-depleted TG-medium containing 1 mM IPTG if required. Cells were collected by centrifugation and re-suspended to an OD_600_ of 0.5 in the same medium. Of these suspensions 50 μl were labeled with H_3_[^32^P]PO_4_ as described before [65]. Phosphorylated proteins were separated by 13% SDS-PAGE and finally analyzed by phospho-imaging (Typhoon FLA-9500; GE Healthcare).

### Labeling of phosphorylated proteins by [^32^P] *in vivo* followed by pull-down assay for their isolation

Cells were grown as described in the section above. Following incubation in TG-medium for 1 h, cultures had a cell density corresponding to OD_600_ = 2–3. Cells equivalent to 5 OD_600_ units were collected by centrifugation and re-suspended in 1.8 ml TG-medium (i.e. OD_600_ = 2.8). Aliquots of the cultures were subjected to SDS-PAGE and Coomassie blue staining or Western blotting to assess proper synthesis of IPTG-inducible proteins. For labeling, 150 μCi H_3_[^32^P]PO_4_ (Hartmann Analytic) were added to the cells and incubation was continued for 45 min at 37°C. If required, 150 μM L-epinephrine (Sigma-Aldrich) was added 5 min prior addition of H_3_[^32^P]PO_4_. Following labeling, cells were pelleted and lysed in 350 μl lysis buffer (100 mM Tris/HCl pH 7.5, 200 mM KCl, 20% sucrose, 1 mM EDTA, 1 mg/ml Lysozyme, 20 μg/ml RNase A) by at least 5 freeze thawing cycles. In case of pulse-chase experiments, the assay was scaled up accordingly, i.e. 20 OD_600_ units cells were collected and re-suspended in 7.2 ml TG-medium containing IPTG and 400 μCi H_3_[^32^P]PO_4_ were added for labeling. Labeling was stopped after 45 min by addition of 40 mM Na_2_HPO_4_ and 20 mM KH_2_PO_4_. Subsequently, 1.8 ml samples were removed at indicated times and subjected to lysis and protein pull-down. For pull-down, crude extracts were cleared by centrifugation (15000 rpm, 1 h, 4°C) and subsequently 500 μl buffer W (100 mM Tris/HCl pH 8.0, 150 mM NaCl, 1 mM EDTA) and 10 μl MagStrepXT magnetic beads (IBA, Germany) were added to the cleared lysates and further incubated for 30 min on ice. The magnetic beads were washed 2× using 500 μl buffer W and finally dissolved in 50 μl Laemmli buffer (62.5 mM Tris/HCl pH 6.8, 5% (v/v) 2-mercaptoethanol, 2% (w/v) SDS, 10% (v/v) glycerol, 0.05% (w/v) bromophenolblue). Dissolved beads (5–10 μl) were loaded on SDS-PAA gels (12.5–15%) and analyzed by phospho-imaging or Western blotting using anti-Strep antiserum (1:20000, Promokine). Loading volumes were adjusted according to protein amounts detected in pilot Western blots. Signal intensities were quantified using software ImageQuant TL 8.1 (GE Healthcare).

### Protein purification

Strep-tagged proteins were purified as described previously [66]. Recombinant proteins were overproduced in strain Z196 using the following plasmids encoding the proteins in parentheses: pDL35 (Strep-PhoB), pYG278 (QseF-Strep), pYG278-D56A (QseF_D56A_-Strep), pYG279 (QseF-NTD-Strep), pYG279-D56A (QseF-NTD_D56A_-Strep) and pYG280 (QseF-CTD-Strep). Bacteria were grown in 100 ml LB to an OD_600_ of ~0.8 and synthesis of proteins was induced by addition of 1 mM IPTG for 1 h. Cells were harvested by centrifugation (20’, 4000 rpm, 4°C), washed in buffer W and disrupted by passage through a French pressure cell. Lysates were cleared by centrifugation (14000 rpm, 1 h, 4°C) and loaded on pre-equilibrated columns containing 100 μl StrepTactin resin (IBA, Germany). Samples were 4× washed using 2 ml buffer W prior to elution with 150 μl buffer E (100 mM Tris/HCl pH 8.0, 150 mM NaCl, 1 mM EDTA, 2.5 mM desthiobiotin). Following addition of 20% glycerol, protein fractions were stored at -20°C until further use.

### Western blotting

Protein samples were dissolved in Laemmli buffer and heated for 5 min at 65°C (for samples containing magnetic beads heat denaturation was omitted). Proteins were separated on 12.5–15% SDS PAA gels and blotted onto a polyvinylidene difluoride (PVDF) membrane (GE Healthcare) by semi-dry blotting for 60–120 min at 2.0 mA/cm^2^. Rabbit polyclonal antisera directed against the 3×FLAG-Tag (Lactan) and the Strep-epitope (Promokine) were used in a dilution of 1:5000 and 1:20000, respectively, containing 3% BSA. The phospho-threonine specific antibody (Cell Signaling Technology) was used in a dilution of 1:2000 containing 5% BSA. Primary S1 antiserum was used in a 1:20000 dilution containing 3% BSA. Secondary goat anti-rabbit IgG antibodies conjugated to alkaline phosphatase (1:100 000; Promega) were used together with the CDP* detection system (Roche Diagnostics) to detect the primary antibodies. The maltose binding protein MalE was detected using recombinant monoclonal mouse anti-MBP antibody (Sigma Aldrich) in a dilution of 1:10000. The primary antibody was detected using a secondary HPR coupled anti-mouse antibody. MalE protein was visualized using the Westar sun ECL system (WESTAR) and a chemiluminescence detector (ChemiDoc, BioRad).

### Protein ligand fishing by StrepTactin affinity chromatography

Ligand fishing experiments were carried out as described previously [4, 66]. Bait plasmids for expression of the Strep-tag only (pBGG237, negative control) or QseG-Strep (pYG191) were introduced into strains Z952 and Z986 carrying *qseE-3xFLAG* or *phoQ-3xFLAG* preys on the chromosome, respectively. Cells were grown in LB to late exponential phase and expression of bait proteins was induced with 1 mM IPTG for one additional hour. Cells were harvested, lysed and proteins were purified by StrepTactin affinity chromatography as described before [66]. Eluates were diluted in Laemmli buffer and separated by SDS-PAGE followed by Western blotting analysis using an anti-FLAG antiserum. For SDS-PAGE 5 μg of the cleared lysates (total extracts) were loaded onto the gel. Output samples were normalized to the eluted QseG-Strep bait protein amount (i.e. 0.5 μg QseG-Strep) in case QseG-Strep was the bait (Fig 7A, lanes 7 and 9). Corresponding volumes of the eluates obtained from the Strep-tag only co-purifications (Fig 7A, lanes 6 and 8) were loaded.

### Bacterial two-hybrid analysis

For monitoring of protein-protein interactions *in vivo*, the BACTH system was used [45, 46]. BACTH relies on reconstitution of activity of the split *Bordetella pertussis* adenylate cyclase toxin. Reconstitution and thus cAMP production occurs through interaction of candidate proteins fused to the separately encoded T18- and T25-fragments of the *B*. *pertussis* toxin. The plasmid-encoded fusion genes are tested in *E*. *coli* strain BTH101, which lacks endogenous adenylate cyclase activity. Interaction can be monitored quantitatively by measuring activity of β-galactosidase, whose synthesis depends on cAMP-CRP. Plasmid pKT25 and pUT18C were used for construction of in-frame fusions of the candidate genes to the 3′ ends of the sequences encoding T25 and T18, respectively. Plasmid pUTM18C is a derivative of pUT18C that allows translocation of the C-terminally fused candidate protein into the periplasm, while the N-terminal T18-fragment remains in the cytoplasm [46]. BTH101 was co-transformed with the plasmids carrying the desired T18 and T25 fusion genes using selection for kanamycin and ampicillin. The resulting transformants were grown at 28°C in selective LB medium containing 1 mM IPTG for inactivation of the Lac repressor and β-galactosidase activities were determined from cells grown to the stationary phase.

### Random mutagenesis of the QseE N-terminus and screen for loss of interaction with QseG

The *qseE* gene was amplified by error prone PCR [67] using primers BG646/BG647 and plasmid pYG199 as template. Three independent reactions were performed and PCR products were digested with PstI and BspHI. The 789 bp DNA fragment carrying the *qseE*-5’ end was isolated and used to replace the corresponding *wild-type* sequence in the BACTH plasmid pYG199. The ligation reactions were introduced into strain BTH101 carrying plasmid pYG242 coding for T18-TM_oppB_-QseG and recombinants were selected at 28°C on LB agar plates containing the required antibiotics, 40 μg/ml X-Gal and 1 mM IPTG. Plasmids were isolated from colonies exhibiting colorless or pale blue phenotypes indicating impaired QseE/QseG interaction and re-introduced into BTH101/pYG242 to confirm persistence and uniformity of the phenotype. Plasmids passing this test were isolated once more and sequenced. Plasmids carrying *qseE* alleles with stop- or frameshift mutations were not further analyzed. Finally, two plasmids (named pYG199_1.6 and pYG199-TM1; S2 Table) were obtained encoding QseE variants with amino acid exchanges.

## Supporting information

S1 Fig QseG localizes to the cell envelope in *E*. *coli* K-12. *E*. *coli* strain Z197 harboring either plasmid pYG191 coding for QseG-Strep (lanes 3, 4, 7, 8) or the isogenic plasmid pBGG237 encoding solely the Strep-peptide (lanes 1, 2, 5, 6) was grown in M9 minimal medium supplemented with maltose to allow for synthesis of maltose binding protein (MBP a.k.a. MalE). Cells were harvested in exponential (lanes 1, 3, 5, 7) and stationary growth phase (lanes 2, 4, 6, 8) and aliquots were used for preparation of total protein extracts, analyzed in lanes 1–4. The remaining cells were subjected to the TSE fractionation protocol and 6.25 μg of the resulting periplasmic extracts (lanes 5–8) were separated alongside the whole cell extracts (lanes 1–4) by SDS-PAGE and analyzed by Western blotting using α-Strep antiserum (top panel), α-S1 antiserum (second panel from top), α-MBP antibody (third panel from top) and by Coomassie blue staining (bottom panel).
(EPS)

S2 Fig Growth curves for the experiments presented in Fig 1B (A) and Fig 2B (B). Experimental conditions are described in the legends to Figs 1B and 2B, respectively.
(EPS)

S3 Fig Expression of the *glmY’-lacZ* fusion in strain MG1655 derivatives. β-Galactosidase activities of MG1655 derivatives carrying a transcriptional *glmY’-lacZ* fusion on the chromosome. The following strains were employed: Z741 (wild-type), Z981 (Δ*qseG*) and Z981 carrying either the empty expression plasmid pKESK23 or plasmid pYG220 encoding *qseG* under *P*_*tac*_ control. Cells were grown in LB to the exponential and stationary growth phase and the β-galactosidase activities were determined.
(EPS)

S4 Fig Complementation of the chromosomal *qseG* deletion using an arabinose-inducible *qseG* expression plasmid. The measurements shown in Fig 1C were partially repeated, but the arabinose-inducible *qseG* expression plasmid pBGG225 rather than the IPTG-inducible *qseG* vector pYG220 was used for complementation. β-Galactosidase activities were determined from exponentially growing cells of the following strains and transformants (left to right): Z179, Z477, Z477/pBGG225, Z190, Z449, Z449/pBGG225.
(EPS)

S5 Fig QseG levels are limiting for *glmY* expression. The low copy plasmids pYG222 carrying *qseG* under *P*_*Ara*_ control and the isogenic empty expression vector pBGG418 were tested in strain Z449, respectively. Strain Z449 carries a deletion of the chromosomally encoded *qseG* gene and a *glmY’-lacZ* reporter fusion that is exclusively transcribed from the σ^54^-dependent promoter (-10 sequence mutated). The bacteria were grown in LB containing either 0.2% glucose for tight repression (first two columns) or the following L-arabinose concentrations for gradual induction of the *P*_*Ara*_ promoter: 0.025%, 0.05%, 0.1%, 0.2%, 0.4%, 0.8%, 1.6%. Subsequently, the β-galactosidase activities were determined from exponentially growing cells.
(EPS)

S6 Fig Expression of transcriptional *qseE’-lacZ* reporter fusions in the stationary growth phase. The same transformants as described in Fig 3A were used, but as a difference β-galactosidase activities were determined from cells in the stationary growth phase.
(EPS)

S7 Fig The QseG-3×FLAG protein is functional. Strain Z951 carries a *qseG*-3×FLAG allele at the natural *qseG* locus. In addition, the *glmY’-lacZ* reporter fusion is integrated in the *λattB* site on the chromosome. The β-galactosidase activities produced by this strain in the exponential and stationary growth phases (middle columns) were compared to the activities produced by isogenic strains lacking the 3×FLAG fusion (strain Z197, columns left) or *qseG* (strain Z477, columns right).
(EPS)

S8 Fig Plasmid-encoded QseG-Strep protein complements a chromosomal *qseG* deletion. β-Galactosidase activities produced by strains Z197 (wild-type, column 1) and Z477 (Δ*qseG*, columns 2–4), which carry a *glmY’-lacZ* reporter fusion on the chromosome. Strain Z477 harbored the following plasmids: pKESK23 (empty plasmid, column 2), pYG220 (*qseG*, column 3) and pYG191 (*qseG-strep*, column 4). Enzyme activities were determined from exponentially growing cells.
(EPS)

S9 Fig Role of localization signals for QseG activity. Complementation analysis assessing the ability of QseG variants to activate *glmY* expression. The following plasmids encoding the indicated QseG variants under *P*_*tac*_ control were introduced into the *ΔqseG* mutant Z477 carrying a *glmY’-lacZ* reporter fusion on the chromosome and the β-galactosidase activities were determined from cells grown to exponential phase (top panel) as well as to stationary phase (bottom panel): pYG220 (wt-QseG; column 3), pYG226 (QseG Δ1–25; column 4), pYG225 (QseG-C26A; column 5), pYG227 (QseG-V27D; column 6), pKESK23 (empty vector, column 7). In addition, the non-transformed strains Z197 (*wild-type*) and Z477 (*ΔqseG*) were employed for comparison (columns 1 and 2).
(EPS)

S10 Fig Sequence alignment of QseE proteins from various *Enterobacteriaceae*. Fully conserved amino acid residues are highlighted in red, while residues conserved in at least half of the species are in blue. Functional domains and important amino acid residues are indicated by horizontal lines and vertical arrows, respectively. Sequences were compiled from the following species (accession numbers are in parentheses): *Escherichia coli* MG1655 (NP_417051.2), *Escherichia coli* O157:H7 str. TW14359 (PJR31962.1), *Shigella dysenteriae* 155–74 (EGI94861.1), *Escherichia albertii* TW07627 (WP_000832932.1), *Citrobacter freundii* (WP_043017212.1), *Salmonella enterica* subsp. *enterica* serovar Typhimurium str. LT2 (NP_461499.1), *Klebsiella pneumoniae* ATCC 25955 (WP_009485444.1), *Serratia marcescens* subsp. *marcescens* (CDG13606.1), *Yersinia pestis* CO92 (AJJ86485.1), *Yersinia pseudotuberculosis* YPIII (WP_002216114.1), *Erwinia tasmaniensis* Et1/99 (CAO96054.1), *Proteus mirabilis* (WP_103388845.1). The alignment was compiled using the AlignX tool of software Vector NTI^™^ 11.0.
(EPS)

S11 Fig Pull-down assay demonstrating that the S58N mutation in QseE decreases interaction with QseG. Strain Z970 was used, which carried two compatible plasmids encoding the proteins as follows: pYG318 (QseE-3×FLAG; lanes 2, 3, 6, 7), pYG318-S58N (QseE-S58N-3×FLAG; lanes 4, 5, 8, 9), pMM10 (Strep-tag only; lanes 2, 4, 6, 8), pYG319 (QseG-Strep; lanes 3, 5, 7, 9). The various transformants were grown in 50 ml LB and expression of plasmid-borne alleles was induced by addition of 1 mM IPTG and 0.2% arabinose, respectively. Following 3 h growth, cultures were harvested by centrifugation and re-suspended in 3 ml lysis buffer (100 mM Tris/HCl pH 7.5, 200 mM KCl, 20% sucrose, 1 mM EDTA, 1 mg/ml lysozyme). Cells were disrupted by one passage through a French pressure cell and the resulting lysates were cleared by centrifugation (60 min, 4°C, 20.000×g). The cleared lysates were incubated with 10 μl (5% solution) pre-equilibrated MagStrep “type3” XT beads (IBA Lifescience) for 60 min at 4°C in an end-over-end shaker. Magnetic beads were collected using a magnet and washed 4× using 500 μl buffer W each and finally re-suspended in 50 μl 1×Laemmli loading dye. 4 μl of the cleared lysates (total extracts) and 20 μl of each eluate were analyzed by SDS-PAGE and Western blotting. In lane 1, strain Z197 carrying plasmid pBGG237 was employed as negative control.
(EPS)

S12 Fig The residual activation potential of the QseE-S58N mutant depends on QseG. β-Galactosidase activities of *wild-type* (Z197) and Δ*qseG* (Z477) strains carrying a transcriptional *glmY’-lacZ* fusion on the chromosome. In addition, the following plasmids encoding the indicated proteins were present in strain Z477: pKESK23 (empty vector), pYG221 (wild-type QseE), pYG221-S58N (QseE-S58). Enzyme activities were determined from cells in the exponential as well as stationary growth phase.
(EPS)

S13 Fig Sequence alignment of QseG proteins from various *Enterobacteriaceae*. Fully conserved amino acid residues are highlighted in red, while residues conserved in at least half of the species are in blue. The signal peptide and location of the lipobox are indicated. Sequences were compiled from the following species (accession numbers are in parentheses): *Escherichia coli* MG1655 (NP_417050.1), *Escherichia coli* O157:H7 str. TW14359 (ACT73265.1), *Shigella dysenteriae* 155–74 (EGI94769.1), *Escherichia albertii* TW07627 (WP_024164742.1), *Citrobacter freundii* (WP_044714650.1), *Salmonella enterica* subsp. *enterica* serovar Typhimurium str. LT2 (NP_461498.1), *Klebsiella pneumoniae* ATCC 25955 (WP_002914033.1), *Serratia marcescens* subsp. *marcescens* (CDG13605.1), *Yersinia pestis* CO92 (YP_002347846.1), *Yersinia pseudotuberculosis* YPIII (WP_072085126.1), *Erwinia tasmaniensis* Et1/99 (CAO96055.1), *Proteus mirabilis* (WP_049221196.1). The alignment was compiled using the AlignX tool of software Vector NTI^™^ 11.0.
(EPS)

S14 Fig No role of phosphate and sulfate for transcription of *glmY*. Strain Z197 carrying a *glmY’-lacZ* fusion on the chromosome was grown in MOPS medium (40 mM MOPS pH 7.4, 4 mM Tricine, 100 μM FeCl_3_, 9.5 mM NH_4_Cl, 0.5 μM CaCl_2_, 0.53 mM MgCl_2_, 50 mM NaCl, 1% glucose, 40 μg/ml proline, 1 μg/ml thiamine) in absence (column 1) or presence of phosphate (columns 2–3) or sulfate salts (columns 4–5) for 24 h to an OD_600_ of ~0.9 and subsequently the β-galactosidase activities were measured.
(EPS)

S15 Fig QseB has no role for expression of *glmY* in *E*. *coli* K-12. β-Galactosidase activities produced by strains Z197 (*wild-type*) and Z891 (*ΔqseB*) carrying a *glmY’-lacZ* reporter fusion in the *λattB* site on the chromosome. The bacteria were grown in LB and the β-galactosidase activities were determined at the indicated times during growth.
(EPS)

S16 Fig The QseB/QseC TCS does not affect activity of the σ^54^-promoter directing expression of *glmY*. β-Galactosidase activities produced by strains Z190 (*wild-type*), Z401 (*ΔqseC*), Z890 (*ΔqseB*) and Z196 (*ΔqseF*) carrying a *glmY’-lacZ* reporter fusion transcribed from the σ^54^-promoter (i.e. the -10 sequence of the σ^70^-promoter was mutated) in the *λattB* site on the chromosome. The bacteria were grown in LB and the β-galactosidase activities were determined from cells grown to the exponential (left) as well as to the stationary growth phase (right).
(EPS)

S17 Fig The serine/threonine kinases SrkA (a.k.a. YihE) and YeaG are dispensable for phosphorylation of the QseF-CTD *in vivo*. StrepTactin pull-down assay after metabolic ^32^P labeling. Strains Z196, Z1044 (Δ*srkA*) and Z1045 (Δ*yeaG*) were addressed, which lacked the endogenous *qseF* gene. Plasmid pYG280 encoding QseF-CTD-Strep was introduced into these strains and the transformants were grown to an OD_600_ of ~0.5–0.8 prior to addition of 1 mM IPTG and metabolic [^32^P] labeling. Induction of QseF-CTD-Strep synthesis was assessed by SDS-PAGE and Coomassie blue staining of total protein extracts (left). Pull-down fractions containing QseF-CTD-Strep were analyzed by Western Blotting (middle) using an antibody directed against the Strep-tag and autoradiography (right).
(EPS)

S1 Table Strains used in this study. (DOCX)

S2 Table Plasmids used in this study. (DOCX)

S3 Table Oligonucleotides used in this study. (DOCX)

S1 Text Construction of plasmids and site-directed mutagenesis. (DOCX)
