# Supplementary material for: Interaction of lipoprotein QseG with sensor kinase QseE in the periplasm controls the phosphorylation state of the two-component system QseE/QseF in Escherichia coli
Source: PLoS Genet. 2018 Jul 24;14(7):e1007547. doi: 10.1371/journal.pgen.1007547 (PMC6075780; doi:10.1371/journal.pgen.1007547)
Supplement: S1 Table — (DOCX) [file pgen.1007547.s018.docx]

**S1 Table**. Strains used in this study

| Name | Genotype | Reference |
| --- | --- | --- |
| BTH101 | F^-^ *cya-99* *araD139 galE15 galK16 rpsL1* (Str^R^) *hsdR2 mcrA1 mcrB1* | [[1](#_ENREF_1)] |
| JW1772 | F^-^*,* Δ*(araD-araB)567,* Δ*lacZ4787(::rrnB-3), λ^-^,* Δ*yeaG::kan, rph-1,* Δ*(rhaD-rhaB)568, hsdR51*4 | [[2](#_ENREF_2)] |
| JW2993 | F^-^*,* Δ*(araD-araB)567,* Δ*lacZ4787(::rrnB-3), λ^-^,* Δ*qseB723::kan, rph-1,* Δ*(rhaD-rhaB)568, hsdR51*4 | [[2](#_ENREF_2)] |
| JW2994 | F^-^*,* Δ*(araD-araB)567,* Δ*lacZ4787(::rrnB-3), λ^-^,* Δ*qseC724::kan, rph-1,* Δ*(rhaD-rhaB)568, hsdR51*4 | [[2](#_ENREF_2)] |
| JW3350 | F^-^*,* Δ*(araD-araB)567,* Δ*lacZ4787(::rrnB-3), λ^-^,* Δ*dam-722::kan, rph-1,* Δ*(rhaD-rhaB)568, hsdR51*4 | [[2](#_ENREF_2)] |
| JW3831 | F^-^*,* Δ*(araD-araB)567,* Δ*lacZ4787(::rrnB-3), λ^-^,* Δ*srkA::kan, rph-1,* Δ*(rhaD-rhaB)568, hsdR51*4 | [[2](#_ENREF_2)] |
| R1279 | CSH50 Δ(*pho-bgl*)201 Δ(*lac-pro*) *ara thi* | [[3](#_ENREF_3)] |
| S4197 | MG1655 *rph^+^ ilvG^+^ ΔlacZ* | [[4](#_ENREF_4)] |
| Z179 | as R1279, but Δ*qseE* | [[5](#_ENREF_5)] |
| Z190 | as R1279, but *attB::*[*glmY*′(−238 to +22)-*lacZ,* −10 mutated] | [[5](#_ENREF_5)] |
| Z196 | as R1279, but Δ*qseF*, *attB::*[*glmY*′(−238 to +22)-*lacZ,* −10 mutated] | [[5](#_ENREF_5)] |
| Z197 | as R1279, but *attB::*[*glmY*′(−238 to +22)-*lacZ*] | [[5](#_ENREF_5)] |
| Z201 | as R1279, but *attB::*[*glmY*′(−238 to +22)-*lacZ,* −24 mutated] | [[5](#_ENREF_5)] |
| Z206 | as R1279, but Δ*qseF*, *attB::*[*glmY*′(−238 to +22)-*lacZ*] | [[5](#_ENREF_5)] |
| Z401 | as R1279, but Δ*qseC::kan*, *attB::*[*glmY*′(−238 to +22)-*lacZ,* −10 mutated] | T4GT7 (JW2994)→Z190; this work |
| Z448 | as R1279, but Δ*qseG*::*cat*, *attB::*[*glmY*′(−238 to +22)-*lacZ*] | PCR BG767+BG768→Z197; this work |
| Z449 | as R1279, but Δ*qseG*, *attB::*[*glmY*′(−238 to +22)-*lacZ,* −10 mutated] | Z463 cured from *cat*; this work |
| Z463 | as R1279, but Δ*qseG*::*cat*, *attB::*[*glmY*′(−238 to +22)-*lacZ,* −10 mutated] | T4GT7 (Z448)→Z190; this work |
| Z464 | as R1279, but Δ*qseG*::*cat*, *attB::*[*glmY*′(−238 to +22)-*lacZ,* −24 mutated] | T4GT7 (Z448)→Z201; this work |
| Z477 | as R1279, but Δ*qseG*, *attB::*[*glmY*′(−238 to +22)-*lacZ*] | Z448 cured from *cat*; this work |
| Z492 | as R1279, but Δ*qseG*, *attB::*[*glmY*′(−238 to +22)-*lacZ,* −24 mutated] | Z464 cured from *cat*; this work |
| Z638 | as S4197, but *phoQ-3xFLAG::kan* | PCR BG902+BG903→S4197; this work |
| Z725 | as S4197, but *qseE-3xFLAG*::*kan* | PCR BG968+BG969→S4197; this work |
| Z741 | as S4197, but *attB::*[*glmY*′(−238 to +22)-*lacZ*] | pBGG201/BamHI→S4197; this work |
| Z890 | as R1279, but Δ*qseB::kan*, *attB::*[*glmY*′(−238 to +22)-*lacZ,* −10 mutated] | T4GT7 (JW2993)→Z190; this work |
| Z891 | as R1279, but Δ*qseB*::*kan*, *attB::*[*glmY*′(−238 to +22)-*lacZ*] | T4GT7 (JW2993)→Z197; this work |
| Z918 | as R1279, but *qseE-3xFLAG*::*kan*, *attB::*[*glmY*′(−238 to +22)-*lacZ*] | T4GT7 (Z725)→Z197; this work |
| Z919 | as R1279, but Δ[*qseG*, *qseF*]::*cat* | PCR BG767+BG1301→R1279; this work |
| Z920 | as R1279, but Δ[*qseG*, *qseF*] | Z919 cured from *cat*; this work |
| Z921 | as R1279, but Δ[*qseG*, *qseF*]::*cat*, *attB::*[*glmY*′(−238 to +22)-*lacZ*] | PCR BG767+BG1301→Z197; this work |
| Z922 | as R1279, but Δ[*qseG*, *qseF*], *attB::*[*glmY*′(−238 to +22)-*lacZ*] | Z921 cured from *cat*; this work |
| Z923 | as R1279, but *qseG-3xFLAG*::*kan* | PCR BG1305+BG1306→R1279; this work |
| Z924 | as R1279, but *qseG-3xFLAG* | Z923 cured from *kan*; this work |
| Z951 | as R1279, but *qseG-3xFLAG*, *attB::*[*glmY*′(−238 to +22)-*lacZ*] | pBGG201/BamHI→Z924; this work |
| Z952 | as R1279, but *qseE-3xFLAG*, *attB::*[*glmY*′(−238 to +22)-*lacZ*] | Z918 cured from *kan*; this work |
| Z955 | as R1279, but Δ[*qseG*, *qseF*], *attB::*[*glmY*′(−238 to +22)-*lacZ*, −10 mutated] | pBGG209/BamHI→Z920; this work |
| Z966 | as R1279, but Δ*qseE*::*cat*, *attB::*[*glmY*′(−238 to +22)-*lacZ*] | PCR BG969+BG1390→Z197; this work |
| Z970 | as R1279, but Δ*qseE*, *attB::*[*glmY*′(−238 to +22)-*lacZ*] | Z966 cured from *cat*; this work |
| Z980 | as S4197, but Δ*qseG*::*cat, attB::*[*glmY*′(−238 to +22)-*lacZ*] | T4GT7 (Z448)→Z741; this work |
| Z981 | as S4197, but Δ*qseG, attB::*[*glmY*′(−238 to +22)-*lacZ*] | Z980 cured from *cat*; this work |
| Z984 | as R1279, but *phoQ-3xFLAG::kan* | T4GT7 (Z638*)*→R1279; this work |
| Z986 | as R1279, but *phoQ-3xFLAG* | Z984 cured from *kan*; this work |
| Z1044 | as R1279, but Δ*qseF*, *attB::*[*glmY*′(−238 to +22)-*lacZ,* −10 mutated], Δ*srkA::kan* | T4GT7 (JW3831*)*→Z196; this work |
| Z1045 | as R1279, but Δ*qseF*, *attB::*[*glmY*′(−238 to +22)-*lacZ,* −10 mutated], Δ*yeaG::kan* | T4GT7 (JW1772*)*→Z196; this work |
| Z1116 | as R1279, but Δ*qseG*::*cat* | T4GT7 (Z448*)*→R1279; this work |
| Z1117 | as R1279, but Δ*qseG* | Z1116 cured from *cat*; this work |

**References**

1. Karimova G, Pidoux J, Ullmann A, Ladant D. A bacterial two-hybrid system based on a reconstituted signal transduction pathway. Proc Natl Acad Sci U S A. 1998;95(10):5752-6. PubMed PMID: 9576956.

2. Baba T, Ara T, Hasegawa M, Takai Y, Okumura Y, Baba M, et al. Construction of *Escherichia coli* K-12 in-frame, single-gene knockout mutants: the Keio collection. Mol Syst Biol. 2006;2:1-11. PubMed PMID: 16738554.

3. Görke B, Rak B. Catabolite control of *Escherichia coli* regulatory protein BglG activity by antagonistically acting phosphorylations. Embo J. 1999;18(12):3370-9. PubMed PMID: 10369677.

4. Venkatesh GR, Kembou Koungni FC, Paukner A, Stratmann T, Blissenbach B, Schnetz K. BglJ-RcsB heterodimers relieve repression of the *Escherichia coli bgl* operon by H-NS. J Bacteriol. 2010;192(24):6456-64. PubMed PMID: 20952573.

5. Reichenbach B, Göpel Y, Görke B. Dual control by perfectly overlapping sigma 54- and sigma 70- promoters adjusts small RNA GlmY expression to different environmental signals. Mol Microbiol. 2009;74(5):1054-70. PubMed PMID: 19843219.
