# Supplementary material for: Interaction of lipoprotein QseG with sensor kinase QseE in the periplasm controls the phosphorylation state of the two-component system QseE/QseF in Escherichia coli
Source: PLoS Genet. 2018 Jul 24;14(7):e1007547. doi: 10.1371/journal.pgen.1007547 (PMC6075780; doi:10.1371/journal.pgen.1007547)
Supplement: S2 Table — (DOCX) [file pgen.1007547.s019.docx]

**S2 Table**. Plasmids used in this study

| Name | Relevant structure^a^ | Reference |
| --- | --- | --- |
| pBAD18-cm | *P_ara_*, MCS 2, *cat*, *ori* pBR322 | [[1](#_ENREF_1)] |
| pBGG164 | *strep-rapZ* under *P_tac_* control in pKES170 | [[2](#_ENREF_2)] |
| pBGG201 | *λattP, aadA,* [*glmY’*(-238 to +22)*-lacZ*]*, kan, ori* p15A | [[3](#_ENREF_3)] |
| pBGG209 | *λattP, aadA,* [*glmY’*(-238 to +22)*-lacZ, -10* mutated]*, kan, ori* p15A | [[3](#_ENREF_3)] |
| pBGG224 | *qseE* with cognate *RBS* under *P_Ara_* control in pBAD18-cm | [[3](#_ENREF_3)] |
| pBGG225 | *qseG* with cognate *RBS* under *P_Ara_* control in pBAD18-cm | this work |
| pBGG237 | *Strep-tag* under *P_tac_* control in pKES170 | [[2](#_ENREF_2)] |
| pBGG273 | transcriptional fusion of *qseE*’ (-70 to +107) to *lacZ* in pKES15 | this work |
| pBGG274 | transcriptional fusion of *qseE*’ (-480 to +107) to *lacZ* in pKES15 | this work |
| pBGG354 | transcriptional fusion of *qseE*’ (-70 to +266) to *lacZ* in pKES15 | this work |
| pBGG355 | transcriptional fusion of *qseE*’ (-480 to +266) to *lacZ* in pKES15 | this work |
| pBGG389 | *qseF* with cognate *RBS* under *P_Ara_* control in pBAD33 | [[4](#_ENREF_4)] |
| pBGG398 | as pBGG389, but *qseF* with D56A exchange | [[4](#_ENREF_4)] |
| pBGG399 | as pBGG389, but *qseF* with D56E exchange | [[4](#_ENREF_4)] |
| pBGG418 | *araC, P_Ara_, sacB-RBS,* MCS, *cat, ori* pSC101 | [[5](#_ENREF_5)] |
| pBGG427 | *ptsN* under *P_Ara_* control in pBGG418 | [[5](#_ENREF_5)] |
| pBR-plac | IPTG inducible artificial P_LlacO-1_ promoter in pBR322; allows to starts sRNA transcription at authentic +1 position when cloned between AatII/EcoRI | [[6](#_ENREF_6)] |
| pDL35 | *strep-phoB* under *P_tac_* control in pKES170 | this work |
| pKES15 | vector for transcriptional *lacZ* fusions; *bgl‘-lacZ*, *neo*, *ori* p15A | [[7](#_ENREF_7)] |
| pKES168 | *lacI^q^, P_tac_, T7gene10-RBS,* NdeI, XbaI, *rrnBT1/T2, neo, ori* p15A | [[5](#_ENREF_5)] |
| pKES170 | *lacI^q^, P_tac_, T7gene10-RBS,* NdeI, XbaI, *rrnBT1/T2, bla, ori* pBR322 | [[8](#_ENREF_8)] |
| pKESK23 | *lacI^q^, P_tac_*::MCS*, neo, attP, aadA, ori* p15A | [[2](#_ENREF_2)] |
| pKT25 | *P_lac_*::*cyaA* [*1-224*] (T25), MCS, *neo*, *ori* p15A | [[9](#_ENREF_9)] |
| pKT25-zip | encodes T25-GCN4 leucine zipper fusion in pKT25 | [[9](#_ENREF_9)] |
| pKT25-TM-zip | encodes T25-TM_oppB_-GCN4 leucine zipper fusion in pKT25 | [[10](#_ENREF_10)] |
| pMM10 | *lacI^q^, P_tac_:: streptag, MCS, neo, ori* p15A | [[5](#_ENREF_5)] |
| pUT18C | *P_lac_*::*cyaA* [*225-399*] (T18), MCS, *bla*, *ori* ColEI | [[9](#_ENREF_9)] |
| pUT18C-zip | encodes T18-GCN4 leucine zipper fusion in pUT18C | [[9](#_ENREF_9)] |
| pUT18C-TM-zip | encodes T18-TM_oppB_-GCN4 leucine zipper fusion in pUT18C | [[10](#_ENREF_10)] |
| pUTM18C | *P_lac_::cyaA (T18)-TM_oppB_, MCS, bla, ori ColEI* | [[10](#_ENREF_10)] |
| pVK1 | encodes T18-PhoQ in pUT18C | this work |
| pVK2 | encodes T18-CpxA in pUT18C | this work |
| pYG83 | *glmY* in pBR-plac | [[11](#_ENREF_11)] |
| pYG89 | *qseF* with cognate *RBS* under *P_tac_* control in pKESK23 | this work |
| pYG90 | as pYG89, but *qseF* with D56E exchange | this work |
| pYG93 | as pYG89, but *qseF* with D56A exchange | this work |
| pYG191 | *qseG-strep* with *T7gene10-RBS* under *P_tac_* control in pKES170 | this work |
| pYG196 | encodes T18-QseG [Δ 1-25] in pUT18C | this work |
| pYG198 | as pBGG225, but *qseG* with C26A exchange | this work |
| pYG199 | encodes T25-QseE in pKT25 | this work |
| pYG199_1.6 | encodes T25-QseE-S58N in pKT25 | this work |
| pYG199_TM1 | encodes T25-QseE-M5 (F19L, L21H, I22R, L23P, L24P) in pKT25 | this work |
| pYG220 | *qseG* with cognate *RBS* under *P_tac_* control in pKESK23 | this work |
| pYG221 | *qseE* with cognate *RBS* under *P_tac_* control in pKESK23 | this work |
| pYG221-H259A | as pYG221, but *qseE* with H259A exchange | this work |
| pYG221-S58N | as pYG221, but *qseE* with S58N exchange | this work |
| pYG221-TM1 | as pYG221, but *qseE* with F19L, L21H, I22R, L23P, L24P exchanges | this work |
| pYG222 | *qseG* with cognate *RBS* under *P_Ara_* control in pBGG418 | this work |
| pYG223 | as pBGG225, but *qseG* Δ[codon 1-25] | this work |
| pYG224 | as pBGG225, but *qseG* with V26D exchange | this work |
| pYG225 | as pYG220, but *qseG* with C26A exchange | this work |
| pYG226 | as pYG220, but *qseG* Δ[codon 1-25] | this work |
| pYG227 | as pYG220, but *qseG* with V27D exchange | this work |
| pYG228 | *qseF* with *T7gene10-RBS* under *P_tac_* control in pKES168 | this work |
| pYG229 | *qseF*-D56A with *T7gene10-RBS* under *P_tac_* control in pKES168 | this work |
| pYG242 | encodes T18-TM_oppB_-QseG [Δ 1-25] in pUTM18C | this work |
| pYG246 | encodes T18-QseE in pUT18C | this work |
| pYG248 | encodes T25-CpxA in pKT25 | this work |
| pYG250 | encodes T25-PhoQ in pKT25 | this work |
| pYG253 | *qseF* with *T7gene10-RBS* under *P_tac_* control in pKES170 | this work |
| pYG254 | *qseF*-D56A with *T7gene10-RBS* under *P_tac_* control in pKES170 | this work |
| pYG255 | encodes T25-QseE (aa 1-250)-GCN4 leucine zipper fusion in pKT25 | this work |
| pYG256 | encodes T18-QseE (aa 1-250) in pUT18C | this work |
| pYG257 | encodes T18-QseE (aa 1-250)-GCN4 leucine zipper fusion in pUT18C | this work |
| pYG259 | encodes T25-QseE (aa 1-250) in pKT25 | this work |
| pYG268 | *qseF-strep* with cognate *RBS* under *P_tac_* control in pKES170 | this work |
| pYG269 | *qseF-strep* with cognate *RBS* under *P_tac_* control in pKES168 | this work |
| pYG269-D56A | *qseF-*D56A*-strep* with cognate *RBS* under *P_tac_* control in pKES168 | this work |
| pYG278 | *qseF-strep* with *T7gene10-RBS* under *P_tac_* control in pKES170 | this work |
| pYG278-D56A | *qseF-*D56A-*strep* with *T7gene10-RBS* under *P_tac_* control in pKES170 | this work |
| pYG279 | *qseF*-NTD-*strep* under *P_tac_* control in pKES170 | this work |
| pYG279-D56A | *qseF*-NTD-D56A*-strep* under *P_tac_* control in pKES170 | this work |
| pYG280 | *qseF*-CTD-*strep* under *P_tac_* control in pKES170 | this work |
| pYG318 | *qseE-3×FLAG* under *P_Ara_* control in pBAD18-cm | this work |
| pYG318-S58N | as pYG318, but *qseE* with S58N exchange | this work |
| pYG319 | *qseG-strep* under *P_tac_* control in pKES168 | this work |

^a^*ori*: origin of replication; *RBS*: ribosomal binding site, MCS: multiple cloning site

**References**

1. Guzman LM, Belin D, Carson MJ, Beckwith J. Tight regulation, modulation, and high-level expression by vectors containing the arabinose P_BAD_ promoter. J Bacteriol. 1995;177(14):4121-30. PubMed PMID: 7608087.

2. Lüttmann D, Göpel Y, Görke B. The phosphotransferase protein EIIA^Ntr^ modulates the phosphate starvation response through interaction with histidine kinase PhoR in *Escherichia coli*. Mol Microbiol. 2012;86:96-110. PubMed PMID: 22812494.

3. Reichenbach B, Göpel Y, Görke B. Dual control by perfectly overlapping sigma 54- and sigma 70- promoters adjusts small RNA GlmY expression to different environmental signals. Mol Microbiol. 2009;74(5):1054-70. PubMed PMID: 19843219.

4. Göpel Y, Lüttmann D, Heroven AK, Reichenbach B, Dersch P, Görke B. Common and divergent features in transcriptional control of the homologous small RNAs GlmY and GlmZ in *Enterobacteriaceae*. Nucleic Acids Res. 2011;39(4):1294-309. PubMed PMID: 20965974.

5. Mörk-Mörkenstein M, Heermann R, Göpel Y, Jung K, Görke B. Non-canonical activation of histidine kinase KdpD by phosphotransferase protein PtsN through interaction with the transmitter domain. Mol Microbiol. 2017;106(1):54-73. Epub 2017/07/18. doi: 10.1111/mmi.13751. PubMed PMID: 28714556.

6. Guillier M, Gottesman S. Remodelling of the *Escherichia coli* outer membrane by two small regulatory RNAs. Mol Microbiol. 2006;59(1):231-47. PubMed PMID: 16359331.

7. Nagarajavel V, Madhusudan S, Dole S, Rahmouni AR, Schnetz K. Repression by binding of H-NS within the transcription unit. J Biol Chem. 2007;282(32):23622-30. PubMed PMID: 17569663.

8. Lüttmann D, Heermann R, Zimmer B, Hillmann A, Rampp IS, Jung K, et al. Stimulation of the potassium sensor KdpD kinase activity by interaction with the phosphotransferase protein IIA^Ntr^ in *Escherichia coli*. Mol Microbiol. 2009;72:978-94.

9. Karimova G, Pidoux J, Ullmann A, Ladant D. A bacterial two-hybrid system based on a reconstituted signal transduction pathway. Proc Natl Acad Sci U S A. 1998;95(10):5752-6. PubMed PMID: 9576956.

10. Ouellette SP, Gauliard E, Antosova Z, Ladant D. A Gateway((R)) -compatible bacterial adenylate cyclase-based two-hybrid system. Environ Microbiol Rep. 2014;6(3):259-67. Epub 2014/07/02. doi: 10.1111/1758-2229.12123. PubMed PMID: 24983530.

11. Göpel Y, Khan MA, Görke B. Domain swapping between homologous bacterial small RNAs dissects processing and Hfq binding determinants and uncovers an aptamer for conditional RNase E cleavage. Nucleic Acids Res. 2016;44(2):824-37. doi: 10.1093/nar/gkv1161. PubMed PMID: 26531825.
