# Supplementary material for: Interaction of lipoprotein QseG with sensor kinase QseE in the periplasm controls the phosphorylation state of the two-component system QseE/QseF in Escherichia coli
Source: PLoS Genet. 2018 Jul 24;14(7):e1007547. doi: 10.1371/journal.pgen.1007547 (PMC6075780; doi:10.1371/journal.pgen.1007547)
Supplement: S3 Table — (DOCX) [file pgen.1007547.s020.docx]

**S3 Table.** Oligonucleotides used in this study

| Primer | Sequence^a^ | Res. Sites | | Position^b^ |
| --- | --- | --- | --- | --- |
| BG260 | agtggctcattcaccgac | |  | *glmY* +1 to +18 |
| BG261 | CTAATACGACTCACTATAGGGAGataaggcggtgcctaactc | |  | *glmY* +150 to +131 |
| BG287 | TGCCTGGCGGCCGTAG | |  | *rrfD* +1 to +16 |
| BG288 | CTAATACGACTCACTATAGGGAGagcctggcagttccctac | |  | *rrfD* +118 to +102 |
| BG377 | GCACGCGTCGACcttttttgtgtctgtaaatcacg | | SalI | *qseE* -480 to -458 |
| BG410 | Ccatcggcgctacggcgtttc | |  | pKES170 1598-1578 |
| BG484 | ctcgtactcatatgagccataaacctgcgc | | NdeI | *qseF* +1 to +19 |
| BG492 | gcGAGCTCaggctattcgcgtctgacgag | | SacI | *qseE* -30 to -10 |
| BG493 | GGCTCTAGATTATTTCGTGTTTTTCGACGACGG | | XbaI | *qseE* +1428 to +1405 |
| BG494 | gcGAGCTCgcccactgggataggctttaag | | SacI | *qseG* -32 to -11 |
| BG495 | GGCTCTAGATTATGGCTCATCAGGAGTGACC | | XbaI | *qseG* +714 to +693 |
| BG541 | GCACGCgtcgaccccggctttgttgtatctgaac | | SalI | *qseE* -70 to -49 |
| BG542 | GCTCTAGACTTTGCCAGGCTTGCCATGC | | XbaI | *qseE* +107 to +88 |
| BG543 | gcGAGCTCgaaggagatatacatATGCGACACATTTTTCAACG | | SacI, NdeI | *qseG* +1 to +20 |
| BG625 | GCGtctagaCTTTGATAAACCTTCGCCAG | | XbaI | *qseE* +266 to +247 |
| BG646 | ttccgcgactcggcgcgc | |  | pKT25 927-944 |
| BG647 | ggggatgtgctgcaaggcg | |  | pKT25 1124-1106 |
| BG764 | CgtcTCTAGATTAAAAGCGGGTTGAAAAACGATATC | | XbaI | *phoB* +690 to +665 |
| BG767 | caacaattacgcccactgggataggctttaagtctggtgaatatgTGTAGGCTGGAGCTGCTTCG | |  | *qseG* -42 to +3 |
| BG768 | TCCCGGATCGTCATCGACCAATAATAAATGCGCAGGTTTATGGCTCATATGAATATCCTCCTTAGTTCCTATTCC | |  | *qseF* +1 to +48 |
| BG878 | GGCTgctagcATGGCGAGACGTATTCTGGTCG | | NheI | *phoB* +1 to +22 |
| BG902 | GGAGGTGATTTTTGGTCGCCAGCATTCTGCGCCGAAAGATGAAGACTACAAAGACCATGACGG | |  | *phoQ* +1416 to +1458 |
| BG903 | GATTATAACGGATGCTTAACGTAATGCGTGAAGTATGGGCATATTTACATATGAATATCCTCCTTAGTTCCTATTCC | |  | *phoQ* +1506 to +1459 |
| BG968 | gtttgtttccgcattgaattaccgtcgtcgaaaaacacgaaaGACTACAAAGACCATGACGG | |  | *qseE* +1384 to +1425 |
| BG969 | GCCACAGCCGTCGGGGCAATAATCGTTGAAAAATGTGTCGCATATGAATATCCTCCTTAGTTCCTATTCC | |  | *qseG* +43 to +4 |
| BG975 | GCCTCTAGAgatgaaaaaattactgcgtctttttttc | | XbaI | *phoQ* +1 to +27 |
| BG976 | CGTCGGTACCTTATTCATCTTTCGGCGCAGAATG | | KpnI | *phoQ* +1461 to +1438 |
| BG979 | GCCTCTAGAgatgataggcagcttaaccgcg | | XbaI | *cpxA* +1 to +21 |
| BG980 | CGTCGGTACCTTAACTCCGCTTATACAGCGGCA | | KpnI | *cpxA* +1374 to +1352 |
| BG1270 | gtgAAACCAGTAACGTTATACG | |  | *lacI* +1 to +22 |
| BG1301 | ATCTGCTAAACGTAACACATAACGCCAATTCATTCCTTGAAATCGTTTGCCATATGAATATCCTCCTTAGTTCCTATTCC | |  | *qseF* +1355 to +1315 |
| BG1302 | GGCTCTAGAG**a**tgAAACGCTGGCCCGTTTTTCCC | | XbaI | *qseE* +2 to +24 |
| BG1303 | cgcGGTACCTTATTTCGTGTTTTTCGACGACG | | KpnI | *qseE* +1428 to +1406 |
| BG1304 | GcctgcagTTAtttttcgaactgcgggtggctccagctagcTGGCTCATCAGGAGTGAC | | PstI, NheI | *qseG* +711 to +694 |
| BG1305 | GAAAAGCCCGCGCCATCCACCCATGAGGTCACTCCTGATGAGCCAgactacaaagaccatgacgg | |  | *qseG* +667 to +711 |
| BG1306 | CGGATCGTCATCGACCAATAATAAATGCGCAGGTTTATGGCTCATATGAATATCCTCCTTAGTTCCTATTCC | |  | *qseF* +45 to +1 |
| BG1309 | gcgTCTAGAGtgcgtgcagaatcacaataagc | | XbaI | *qseG* +76 to +97 |
| BG1310 | cgcGGTACCTTATGGCTCATCAGGAGTGACC | | KpnI | *qseG* +714 to +693 |
| BG1313 | [P]-CTTATTGTGATTCTGCACG**GC**ACCCAGCAATGCCAG | |  | *qseG* +96 to +61 |
| BG1373 | [P]-TTTTTAAGACATTTATCT**GC**TGAATTAAAAACGCCAC | |  | *qseE* +757 to +793 |
| BG1383 | gcGAGCTCGCCCACTGGGATAGGCTTTAAGTCTGGTGAAT**ATG**gtgcagaatcacaataagccagcc | | SacI | *qseG* -32 to +3 φ +79 to +102 |
| BG1384 | [P]-CTTATTGTGATTCTG**AT**CGCAACCCAGCAATGCCAG | |  | *qseG* +96 to +61 |
| BG1385 | CGAAGATCTGAATTCgcccactgggataggctttaag | | BgIII,EcoRI | *qseG* -32 to -11 |
| BG1390 | ttgAAACGCTGGCCCGTTTTTCCCCGCTCATTACGACAACTGGTATGTAGGCTGGAGCTGCTTCG | |  | *qseE* +1 to +45 |
| BG1393 | GGCTCTAGAGAGCTCTCATTCCTTGAAATCGTTTGCATC | | XbaI, SacI | *qseF* +1335 to +1312 |
| BG1450 | tcccCCCGGGGGCGTTGGGATTCCAGCCATG | | XmaI | *qseE* +750 to +731 |
| BG1462 | cgcGGTACCTTAGCGTTGGGATTCCAGCCATG | | KpnI | *qseE* +750 to +731 |
| BG1477 | GGCTgctagcTTCCTTGAAATCGTTTGCATC | | NheI | *qseF* +1332 to +1312 |
| BG1482 | [P]-gatgcccggcgca**a**tgaagcgatgacc | |  | *qseE* +160 to +186 |
| BG1537 | GGCTgctagcTTCATCGGTGGCTGGCGC | | NheI | *qseF* +390 to +373 |
| BG1538 | ctcgtactcatatgCGCTGGCGCGAGGCAATTG | | NdeI | *qseF* +391 to +409 |
| BG1708 | gcgTCTAGActatttatcgtcgtcatctttgtagtcgatatcatgatctttataatcaccgtcatggtctttgtagtcCTGCAGTTTCGTGTTTTTCGACGACGG | | XbaI, PstI | *qseE* +1425 to +1405 |

^a^Restriction sites are underlined; Positions deviating from the wild-type sequence are in bold; [P] indicates 5’-phosphorylation of the oligonucleotide. Sequences complementary to plasmids pKD3 or pSUB11 are underlined by dashed lines; the recognition site for T7 RNA polymerase is underlined by a dotted line. ^b^Positions are relative to the first nucleotide of the respective gene or plasmid.
