# Supplementary material for: Interaction of lipoprotein QseG with sensor kinase QseE in the periplasm controls the phosphorylation state of the two-component system QseE/QseF in Escherichia coli
Source: PLoS Genet. 2018 Jul 24;14(7):e1007547. doi: 10.1371/journal.pgen.1007547 (PMC6075780; doi:10.1371/journal.pgen.1007547)
Supplement: S1 Text — (DOCX) [file pgen.1007547.s021.docx]

**S1 Text. Construction of plasmids and site-directed mutagenesis**

For construction of recombinant plasmids *E. coli* XL1 blue was used as standard cloning strain. Correct structure of recombinant plasmids was verified by colony PCR, analytical digest and DNA sequencing.

**1.** Plasmids for genetic analyses (Figs 1, 2, 8C, S3, S4, S5, S9, S12)

For construction of plasmid pBGG225 carrying *qseG* under *P_Ara_* control, *qseG* was amplified using primers BG494/BG495 and the resulting PCR fragment was inserted between the SacI and XbaI sites on plasmid pBAD18-cm. Plasmids pYG220 encoding *qseG* under *P_tac_* control (Figs 1B, 1C and 2A) and pYG221 encoding *qseE* under *P_tac_* control (Fig 8C) were obtained by ligating the EcoRI-XbaI fragments of plasmids pBGG225 and pBGG224 [[1](#_ENREF_1)] with the EcoRI/XbaI digested plasmid pKESK23, respectively. The derivative plasmids pYG221-H259A, pYG221-S58N and pYG221-TM1 (used in Fig 8C) are isogenic with plasmid pYG221, but carry codon substitutions within the *qseE* gene. Mutations were introduced by combined chain reaction (CCR), which is based on incorporation of a phosphorylated mutagenesis primer by a thermostable DNA ligase (ampligase; Epicentre) into the PCR product during amplification [[2](#_ENREF_2)]. The 5’-phosphorylated mutagenesis primers BG1482 and BG1373 were used in addition to the external primers BG492/BG493 to introduce the mutations leading to the amino acid substitutions S58N and H259A in QseE, respectively. The resulting DNA fragments were digested with SacI and XbaI and used to replace the corresponding *wild-type* fragment in pYG221. Plasmid pYG221-TM1 encoding *qseE-M5* (M5= F19L, L21H, I22R, L23P, L24P) was generated by isolating the 1026 bp *qseE-M5* fragment using the BsrBI/AatII restriction sites in *qseE* and subsequent ligation of the fragment with plasmid pYG221 that was cleaved with AatII and partially digested with BsrBI.

Plasmids pYG225, pYG226 and pYG227 (S9 Fig) are isogenic with plasmid pYG220, but encode QseG-C26A, QseG Δ1-25 and QseG-V27D variants. These *qseG* alleles were first established in plasmid pBAD18-cm and subsequently moved to plasmid pKESK23. For mutation of codons 26 and 27 of *qseG*, CCRs were carried out using plasmid pBGG225 as template, the external primers BG494/BG495 and the mutagenesis primers BG1313 or BG1384. The CCR fragments were subsequently inserted between the SacI and XbaI-sites on plasmid pBAD18-cm resulting in plasmids pYG198 and pYG224, respectively. For construction of the *qseG* allele lacking the first 25 codons, *qseG* Δ1-25 was amplified using oligonucleotides BG1383/BG495 and the PCR fragment was subsequently cloned between the SacI/XbaI-sites on plasmid pBAD18-cm resulting in plasmid pYG223. Subsequently, the *qseG* alleles were isolated from plasmids pYG198, pYG223 and pYG224 by digestion with EcoI and XbaI and inserted between these sites on plasmid pKESK23, resulting in plasmids pYG225, pYG226 and pYG227. Note that the first codon of *qseG* Δ1-25 in plasmid pYG226 (corresponding to codon 26 in full-length *qseG*) was changed from TGC (Cys) to ATG (Met) to allow for translation of the truncated *qseG* variant.

Plasmids pYG89, pYG90 and pYG93 (Fig 2) encoding *qseF*, *qseF-*D56E and *qseF-*D56A, respectively, were derived from sub-cloning of *qseF* alleles from the previously published plasmids pBGG389, pBGG399 and pBGG398 [[3](#_ENREF_3)]. To this end, plasmids pBGG389, pBGG399 and pBGG398 were isolated from strain JW3350 to prevent methylation of the *dam* site overlapping with the XbaI site used for cloning. Thereafter, the plasmids were digested with XbaI and subsequently subjected to partial digestion with EcoRI (5 min, 37°C) to avoid cleavage of the EcoRI site within the *qseF* gene in a fraction of the plasmid molecules, allowing to isolate the DNA fragments carrying the complete *qseF* genes. The latter fragments were inserted between the EcoRI/XbaI sites on plasmid pKESK23 resulting in plasmids pYG89, pYG90 and pYG93, respectively. Plasmid pYG222 (S5 Fig) encoding *qseG* under *P_Ara_* control is a low copy vector with pSC101 origin of replication (isogenic with pBGG418) and was constructed by replacing the BglII-XbaI fragment (comprising gene *ptsN*) in plasmid pBGG427 with a PCR fragment obtained with primers BG1385/BG495.

**2**. Plasmids harboring transcriptional *qseE’-lacZ* reporter gene fusions (Fig 3 and S6 Fig)

For construction of the various transcriptional *qseE’-lacZ* reporter fusions in plasmids pBGG273, pBGG274, pBGG354 and pBGG355, the SalI-XbaI fragment encompassing the *bgl* promoter on plasmid pKES15 was removed and replaced with PCR fragments that were generated using the following primer pairs: BG541/BG542 (*qseE* -70 to +107), BG377/BG542 (*qseE* -480 to +107), BG541/BG625 (*qseE* -70 to +266), BG377/BG625 (*qseE* -488 to +266).

**3.** Plasmids for metabolic [^32^P] labeling of QseF *in vivo* (Fig 4A) and ligand fishing (Fig 7A, S8 Fig, S11 Fig)

Plasmids pYG253 and pYG254 encoding QseF and QseF-D56A under *P_tac_* control were constructed in two steps. First, the *P_tac_-qseF* cassettes were cloned in plasmid pKES168 (*neo*, ori p15A). From there, the *P_tac_-qseF* alleles were moved to plasmid pKES170 (*bla*, ori pBR322) to increase the *qseF* expression level. Plasmids pKES168 and pKES170 are largely identical but have different origins of replications and antibiotic resistance markers. The *qseF* and *qseF-D56A* alleles were amplified by PCR using primers BG484/BG1393 and plasmids pBGG389 and pBGG398 as templates, respectively. The PCR fragments were inserted between the NdeI and XbaI sites in plasmid pKES168 resulting in plasmids pYG228 and pYG229, respectively. Subsequently, the NdeI-XbaI fragments of plasmids pYG228 and pYG229 were isolated and inserted between the NdeI and XbaI sites of plasmid pKES170 resulting in plasmids pYG253 and pYG254.

For construction of the prey plasmid pYG191 producing QseG-Strep for ligand fishing (Fig 7A), the *qseG* gene was amplified by PCR using oligonucleotides BG543/BG1304, which provided the *qseG* gene at its 3’-end with the sequence encoding the Strep-tag. Following digestion with NdeI and PstI, the PCR fragment was used to replace the *strep-rapZ* fusion in plasmid pBGG164.

In the ligand fishing experiment shown in S11 Fig, the prey proteins QseE-3×FLAG and QseE-S58N-3×FLAG were produced from plasmids pYG318 and pYG318-S58N, respectively. For their construction, the respective *qseE* alleles were amplified using oligonucleotides BG492/BG1708 and plasmids pYG221 and pYG221-S58N as templates, respectively. The reverse primer BG1708 added the *3×FLAG* sequence to the 3’ end of *qseE*. Subsequently, the PCR fragments were inserted between the SacI and XbaI sites on plasmid pBAD18-cm. The bait protein QseG-Strep was produced from the compatible plasmid pYG319. It was constructed by insertion of the ApaI/PstI fragment from plasmid pYG191 between the ApaI/PstI sites of plasmid pKES168.

**4.** Plasmids for metabolic [^32^P] labeling and pull-down of Strep-QseF variants (Figs 4B, 5B, 6, 9, S17) and for protein purification (Fig 5C)

Plasmid pYG269 used for the QseF-Strep pull-down assay (Fig 4B) was constructed in two steps. First, plasmid pYG268 was constructed, which carries the *qseF-strep* fusion translated from the cognate *qseF* RBS under *P_tac_* control in plasmid pKES170. Plasmid pYG268 was built by replacing the ApaI-NheI fragment in plasmid pYG191 (comprising the 3’ end of *lacI*, *P_tac_* and *qseG*) with a DNA fragment comprising the 3’ end of *lacI*, *P_tac_* and *qseF*. The latter fragment was generated by PCR using primers BG1270/BG1477 and plasmid pYG89 as template and subsequent digestion with ApaI and NheI. Subsequently, the fragment comprising *lacI-3’*, *P_tac_* and *qseF-strep* was isolated from plasmid pYG268 by ApaI and PstI digestion and used to replace the ApaI-PstI fragment in plasmid pKES168, resulting in plasmid pYG269. To generate the derivative plasmid pYG269-D56A, the *qseF-strep* sequence was mutated by CCR [[2](#_ENREF_2)]. A CCR reaction was carried out using primers BG1270/BG410, the 5’-phosphorylated mutagenesis primer BG685 and plasmid pYG268 as template. The resulting CCR fragment comprising *lacI-3’*, *P_tac_* and the mutated *qseF-strep* allele was digested with ApaI and PstI and inserted between these sites on plasmid pKES168.

Plasmids pYG278 and pYG278-D56A (Fig 5B) contain *qseF-strep* alleles provided with the strong *T7gene10-RBS* under *P_tac_* promoter control in plasmid pKES170. For their construction, plasmid pYG268 was digested with ApaI and AatII, which cleave in the coding sequence of *lacI* and *qseF*, respectively. Subsequently, the fragment was replaced with ApaI-AatII fragments (comprising *lacI*-3’, *P_tac_*, *T7gene10-RBS* and *qseF*-5’) obtained from digestion of plasmids pYG253 and pYG254, respectively. The plasmids coding for the truncated QseF-Strep variants (Figs 5B, 6) were constructed by replacing the NdeI-NheI fragment (comprising *qseG*) in plasmid pYG191 with the respective *qseF* fragments. Thereby, the following plasmid were generated using the primers and templates mentioned in parentheses: pYG279 (*qseF*-NTD-strep; primers BG484/BG1537; template pYG228), pYG279-D56A (*qseF*-NTD-D56A-strep; primers BG484/BG1537; template pYG229) and pYG280 (*qseF*-CTD-strep; primers BG1538/BG1477; template pYG228). The Strep-PhoB protein (Fig 5C) was overproduced with the help of plasmid pDL35 allowing for its purification. Plasmid pDL35 was generated by replacing the NheI-XbaI fragment (comprising *rapZ*) in plasmid pBGG164 with a PCR fragment obtained with primers BG878/BG764.

**5.** Plasmids used for BACTH

For construction of the BACTH plasmids pVK1 and pVK2, genes *phoQ* and *cpxA* were amplified by PCR using the primer pairs BG975/BG976 and BG979/BG980, respectively, and subsequently inserted between the XbaI and KpnI sites on plasmid pUT18C. Subsequently, the XbaI-KpnI fragments of plasmids pVK1 and pVK2 were isolated and inserted between the XbaI and KpnI sites on plasmid pKT25, respectively, resulting in plasmids pYG250 encoding T25-PhoQ and pYG248 encoding T25-CpxA. The BACTH plasmid pYG196 encoding the T18-QseG fusion protein was obtained by amplification of the *qseG* gene using primers BG1309/BG1310 and subsequent insertion of the PCR-fragment between the XbaI and KpnI sites on plasmid pUT18C. Note that within the *T18-qseG* fusion gene, *qseG* lacks the first 25 codons encoding the N-terminal signal sequence. Therefore, the QseG sequence fused to T18 corresponds to the mature form of QseG as present in the periplasm. In order to assess interaction of QseG in the periplasm, the DNA fragment encoding mature QseG (aa 26-237) in plasmid pYG196 was moved between the XbaI/KpnI-sites of plasmid pUTM18C, resulting in BACTH plasmid pYG242. As a difference to pYG196, plasmid pYG242 harbors an in-frame fusion of the sequence encoding the first transmembrane domain (TM1) of the oligopeptide transporter subunit OppB between the *T18* sequence and *qseG* ensuring periplasmic localization of QseG while the T18 domain remains cytosolic. For construction of BACTH plasmid pYG199, *qseE* was amplified by PCR using primers BG1302/BG1303 and inserted between the XbaI and KpnI sites on plasmid pKT25. Subsequently, the *qseE* fragment was isolated by XbaI/KpnI digestion and inserted between the XbaI and KpnI sites on plasmid pUT18C resulting in the BACTH plasmid pYG246.

The BACTH plasmids pYG256 and pYG259 encode the QseE N-terminus (aa 1-250) fused to the sequences coding for the T18- and T25-fragments, respectively. To obtain these plasmids, codons 1-250 of *qseE* were amplified by PCR using primers BG1302/BG1462 and cloned between the XbaI and KpnI sites on plasmids pUT18C and pKT25, respectively. Plasmids pYG255 and pYG257 are similar to plasmids pYG256 and pYG259, but additionally carry the sequence encoding the leucine zipper homo-dimerization domain of Gcn4 (Gcn4-zip) fused in frame to the 3’ end of *qseE* (codons 1-250). For construction of plasmid pYG255, codons 1-250 of *qseE* were amplified by PCR using primers BG1302/BG1450 providing the resulting DNA fragment with XbaI and XmaI restriction sites at the extremities. Subsequently, the PCR fragment was inserted between the XbaI and XmaI sites present between the *orfs* encoding T25 and Gcn4-zip on plasmid pKT25-zip. Finally, the *qseE (codon 1-250)*φ*Gcn4-zip* fusion gene was moved to pUT18C by sub-cloning using the XbaI and EcoRI sites resulting in plasmid pYG257.

**References**

1. Reichenbach B, Göpel Y, Görke B. Dual control by perfectly overlapping sigma 54- and sigma 70- promoters adjusts small RNA GlmY expression to different environmental signals. Mol Microbiol. 2009;74(5):1054-70. PubMed PMID: 19843219.

2. Bi W, Stambrook PJ. Site-directed mutagenesis by combined chain reaction. Anal Biochem. 1998;256(1):137-40. PubMed PMID: 9466810.

3. Göpel Y, Lüttmann D, Heroven AK, Reichenbach B, Dersch P, Görke B. Common and divergent features in transcriptional control of the homologous small RNAs GlmY and GlmZ in *Enterobacteriaceae*. Nucleic Acids Res. 2011;39(4):1294-309. PubMed PMID: 20965974.
